# Expedient Organocatalytic Syntheses of 4-Substituted Pyrazolidines and Isoxazolidines

**DOI:** 10.3390/molecules21121655

**Published:** 2016-12-01

**Authors:** Tarek Yousfi, Alysha Elliott, Messiad Hanane, Rachid Merdes, Albert Moyano

**Affiliations:** 1 Division de Biotechnologie Industrielle, Centre National de Recherche en Biotechnologie, Ali Mendjli Nouvelle Ville, UV 03 BP E73, Constantine 25000, Algeria; t.yousfi@crbt.dz; 2 Laboratoire de Chimie Appliquée, Département de Chimie, Université 8 Mai 1945—Guelma, BP 401, Guelma 24000, Algeria; merdesra@yahoo.fr; 3 Institute for Molecular Biosciences, The University of Queensland, St. Lucia QLD 4072, Australia; a.elliott@imb.uq.edu.au; 4 Département de Génie de l’Environnement, Faculté de Génie des Procédés, Université de Constantine 3, Ali Mendjli Nouvelle Ville, BP72, Constantine 25000, Algeria; messiad_h@yahoo.fr; 5 Departament de Química Inorgànica i Orgànica, Facultat de Química, Universitat de Barcelona, Martí i Franquès 1-11, Barcelona 08028, Catalonia, Spain

**Keywords:** asymmetric catalysis, isoxazolidinones, pyrazolidinones, organocatalysis, hydrazines, hydroxylamines, enals

## Abstract

The efficient organocatalytic synthesis of heterocyclic systems of biological relevance is a subject of growing interest. We have found that the pyrrolidine/benzoic acid-catalyzed reaction of α-substituted propenals such as methacrolein, 2-benzylpropenal and 2-(*n*-hexyl)propenal with activated hydrazines takes place in very good yields (83%–99.6%) under very mild conditions to afford 4-substituted pyrazolidin-3-ols (as diastereomer mixtures); subsequent oxidation with PCC affords the corresponding-4-substituted-3-pyrazolidinones in essentially quantitative yields. In a similar way, 4-substituted isoxazolidinones are obtained with *N*-Cbz-hydroxylamine as a reagent. The use of chiral diarylprolinol trimethylsilyl ethers as catalysts allows the synthesis of several of these compounds in optically active form, in some cases with excellent enantioselectivity (up to 96:4 er). A preliminary evaluation of the biological activity shows that some of these compounds exhibit interesting antibacterial and antifungal activities.

## 1. Introduction

**Figure 1:**
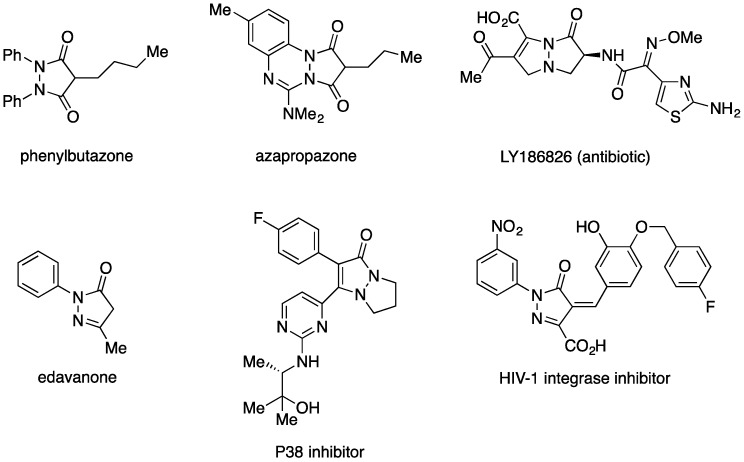
Selected biologically active pyrazolidinones and pyrazolones.

The pyrazolidinone (pyrazolidin-3-one) moiety is an important structural motif that can be found both in natural products and in synthetic compounds with interesting pharmacological activity (analgesic, antibiotic, anticonvulsant, and with inhibitory cyclooxygenase, lipoxygenase, and γ-aminobutyrate transferase activity, among others) [1,2,3,4,5,6,7,8,9,10]. On the other hand, the selective oxidation of pyrazolidinones affords the corresponding pyrazolones (pyrazol-5-ones), a class of compounds that also exhibits very relevant pharmacological properties [11,12,13,14,15,16,17,18]. Figure 1 shows structures of some biologically active pyrazolidinones and pyrazolones.

It is therefore not surprising that efficient and selective syntheses of pyrazolidinone derivatives have been actively pursued [19]. Recently, organocatalytic approaches to these compounds, based either on the amine-catalyzed [20,21] or on the carbene-catalyzed [22] reaction of α,β-unsaturated aldehydes with hydrazine derivatives and subsequent oxidation of the intermediate pyrazolidinols, have been described, enabling their preparation in optically active form [23,24,25]. Although these approaches are highly interesting, all of them suffer from the common limitation that they have been applied only to β-substituted enals, so that they can exclusively lead to 5-substituted-3-pyrazolidinones.

**Scheme 1:**
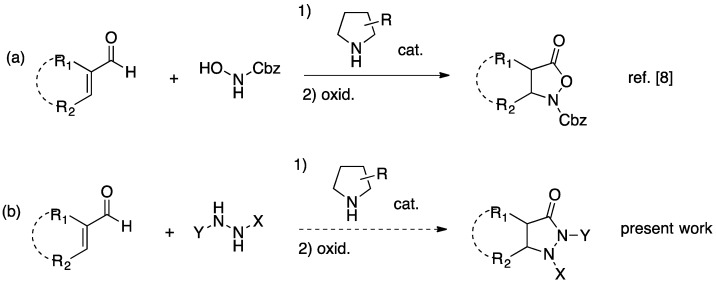
Background (**a**) and goals (**b**) of the present work.

In the course of our studies on the organocatalytic synthesis of α,β-disubstituted-β-amino acids we had found that the pyrrolidine-promoted aza-Michael addition/cyclization of *N*-protected hydroxylamines to acyclic α,β-unsaturated, α,β-disubstituted enals took place in several instances with excellent yields and good diastereoselectivities [26], so that we set out to investigate if the secondary amine-catalyzed reaction of these branched enals with substituted hydrazines, followed by oxidation of the resulting pyrazolidin-3-ols, would provide a general route to 4,5-disubstituted-3-pyrazolidinones (Scheme 1).

## 2. Results and Discussion

### 2.1. Organocatalytic Synthesis of 4-Substituted Pyrazolidinones and Isoxazolidinones

**Scheme 2:**
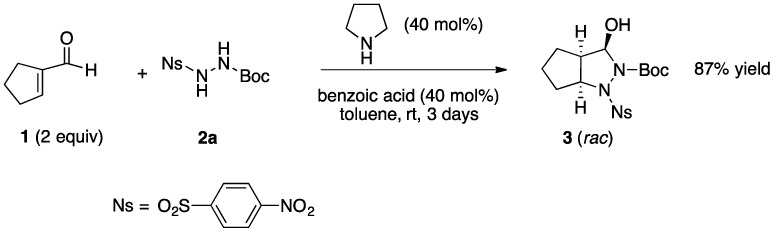
Synthesis of the bicyclic pyrazolidinol **3**.

We began our research by examining the pyrrolidine-catalyzed addition/cyclization of cyclopentene-1-carboxaldehyde (**1**) with 1-Boc-2-(4-nitrobenzenesulfonyl)hydrazine (**2a**) [20] using benzoic acid as a co-catalyst. After some experiments, we found that by using 40 mol % of both pyrrolidine and benzoic acid the expected bicyclic pyrazolidin-3-ol **3** was obtained in excellent yield (87% isolated yield after chromatographic purification) and as a single isomer (dr > 30:1), after three days in toluene at room temperature (r.t.) (Scheme 2).

**Scheme 3:**
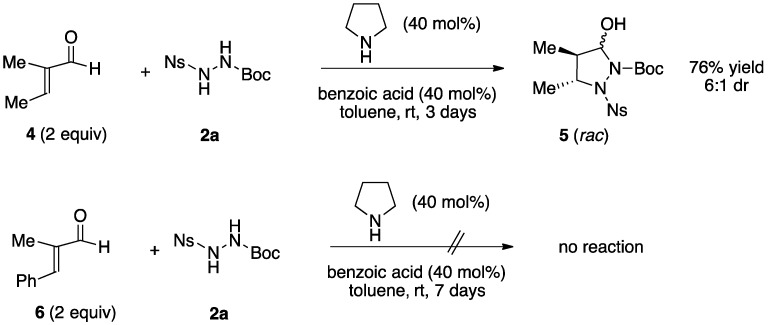
Reaction of hydrazine **2a** with the acyclic α,β-disubstituted enals **4** and **6**.

Next, we tried the same reaction conditions with acyclic α,β-substituted enals. While the reaction with tiglic aldeyhyde ((*E*)-2-methylbutenal, **4**) led to the formation of the pyrazolidinol **5** (76% yield, 6:1 dr), we found that (*E*)-2-methyl-3-phenylbutenal **6** was completely unreactive, no product being detected after 7 days of stirring at r.t. (Scheme 3).

Contrary to what we had observed for the corresponding isoxazolidinols [26], attempted oxidation of both **3** and **5**, either with pyridinium chlorochromate (PCC) or with pyridinium dichromate (PDC), failed to cleanly give the desired pyrazolidinones, so we decided to reduce the steric hindrance of the intermediate pyrazolidinols by suppressing the β-substituent in the starting enal.

**Scheme 4:**
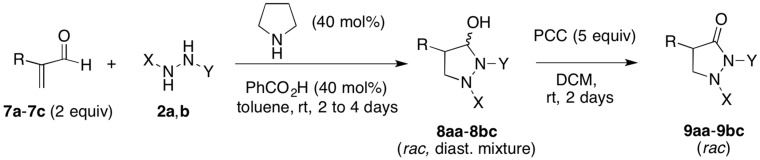
Organocatalytic synthesis of pyrazolidin-3-ones from α-substituted enals.

We were pleased to find that the pyrrolidine/benzoic acid-catalyzed reaction of the α-substituted enals methacrolein (**7a**), 2-benzylpropenal (**7b**), or 2-(*n*-hexyl)propenal (**7c**) with the activated hydrazines **2a** and 1,2-bis(*p*-toluenesulfonyl)hydrazine (**2b**) took place in very good yields (83%–99.6%), affording the 4-substituted pyrazolidin-3-ols **8aa**–**8bc** as diastereomer mixtures; without further purification, oxidation with PCC was performed to afford the corresponding-4-substituted-3-pyrazolidinones **9aa**–**9bc** in essentially quantitative yields (Scheme 4 and Table 1).

As it can be seen, the aza-Michael addition/cyclization step took place in excellent yield and with high diastereoselectivity, irrespective of the nature of the hydrazine (**2a** or **2b**) and of the acrolein substituent (methyl, benzyl, or *n*-hexyl). It should be noted however that when the more hindered α-isopropyl acrylaldehyde (3-methyl-2-methylenebutyraldehyde (**7d**)) was used under the same conditions, no reaction with hydrazine **2a** was observed after 7 days at rt. In our hands, *N,N’*-bis(*tert*-butoxycarbonyl)hydrazine (**2c**), reported to give good yields of pyrazolidin-3-ols upon pyrrolidine-catalyzed reaction with cinnamaldehyde derivatives [21], failed to react with the α-substituted enals **1**, **4**, and **7a**–**7d**.

**Scheme 5:**
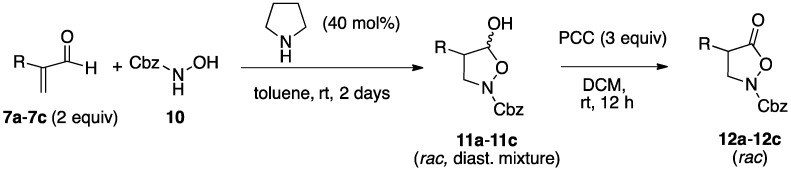
Organocatalytic synthesis of isoxazolidin-5-ones from α-substituted enals.

In the light of these results, we explored the possible use of the α-substituted acroleins **7** as starting materials for the synthesis of 4-substituted isoxazolidin-5-ones, potentially interesting compounds both from the biological point of view and as advanced intermediates towards α-substituted-β-amino acids [26], by the amine-catalyzed reaction with a suitable protected hydroxylamine. To our satisfaction, we found that in fact α-substituted acroleins **7**, with the sole exception of the isopropyl derivative **7d**, reacted smoothly with *N*-Cbz-hydroxylamine **10** (Cbz = benzyloxycarbonyl) to afford the isoxazolidinols **11** in good yields (78%–83% isolated yields) as diastereomer mixtures. Subsequent oxidation of these compounds with PCC took place uneventfully, providing the target 4-substituted isoxazolidin-5-ones **12** (Scheme 5 and Table 2).

**Figure 2:**
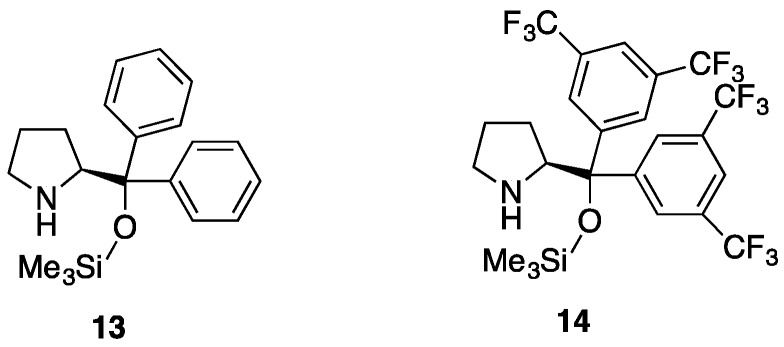
Jørgensen-Hayashi catalysts **13** ((*S*)-diphenylprolinol trimethylsilyl ether) and **14** ((*S*)-bis(3,5-trifluoromethylphenyl)prolinol trimethylsilyl ether) used in the enantioselective synthesis of pyrazolidinones **9**.

Previous studies from our group [26] and those of Córdova [27,28] and Vicario [20] had shown that the use of chiral diarylprolinol-silyl ethers as catalysts led to high enantioselectivities in the aza-Michael/cyclization reactions of β-substituted [20,27,28] and of α,β-disubstituted [26] enals with hydroxylamines or hydrazines. However, α-branched vinyl carbonyls remain a very challenging substrate for this type of reaction and only one successful example of aminocatalytic asymmetric aza-Michael addition to α-substituted vinyl ketones has been reported so far [29,30]. Bearing these precedents in mind, we proceeded to examine the performance of the Jørgensen-Hayashi catalysts **13** and **14** (Figure 2) in the reaction between aldehydes **7a**–**7c** and hydrazines **2a** and **2b** (Table 3), using a 20 mol % of the catalyst together with a 20 mol % of benzoic acid co-catalyst in toluene at r.t. (typical reaction time 3 days). The stereoisomeric mixture of pyrazolidinols **8** was then submitted to oxidation with PCC as before, and the enantiomeric purity of the corresponding pyrazolidinones **9** was determined by chiral HPLC analysis. After 7 days of stirring at rt, no product was observed in the attempted reactions of aldehyde **7c** with hydrazine **2a**, either when using **13** or **14** as catalysts. On the other hand, we could not find satisfactory HPLC conditions for the separation of the enantiomers of **9bb**, so that the enantioselective catalysis for the reaction of aldehyde **7b** with hydrazine **2b** was not attempted.

As it can be seen in Table 3, the enantioselectivity of the reaction depends strongly both in the nature of the reactants and of the catalyst. The highest enantiomeric purities were obtained for the less hindered methyl-substituted enal **7a**, either with hydrazine **2a** (entry 2, 90:10 er, catalyst **14**) or with hydrazine **2b** (entry 7, 96:4 er, catalyst **13**). In any case, these preliminary results demonstrate for the first time the feasibility of the asymmetric synthesis of 4-substituted-3-pyrazolidinones by the organocatalytic aza-Michael/cyclization of activated hydrazines to α-substituted acroleines.

**Scheme 6:**
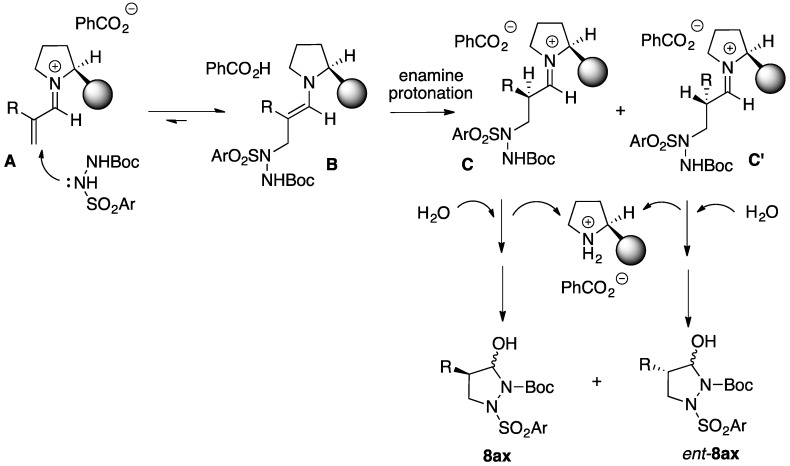
Mechanistic proposal for the Michael addition-cyclization sequence leading to the formation of enantiomerically enriched 4-substituted pyrazolidinols.

A simplified mechanistic proposal for the formation of enantiomerically enriched 4-substituted pyrazolidinols **8aa**–**8ac** is depicted in Scheme 6. While in the formation of the first carbon-nitrogen bond by the aza-Michael reaction of the chiral iminium intermediate **A** (formed from the α-substituted enal **7a**–**c** and the chiral pyrrolidine catalyst **13** or **14**) with the hydrazine **2a** no new chiral center is formed, the protonation of the intermediate enamine **B** can take place on the two diastereotopic faces of the C–C double bond. When using the trifluoromethyl-substituted catalyst **14**, we assume (based on our previous studies on the asymmetric organocatalytic synthesis of isoxazolidines) [26] that when R = Me protonation will take place under kinetic control from the face opposite to the bulky pyrrolidine substituent, leading to the second iminium intermediate **C** as the major diastereomer. Fast, irreversible hydrolysis and cyclization of this intermediate would predominantly give the (4*R*) enantiomer of **8aa**. When the steric bulk of the R substituent is increased (**7b** or **7c**), the formation of the diastereomeric iminium ion **C’** will be relatively favoured (in this intermediate the steric interactions of the α-substituent R with the pyrrolidine substituent are minimized), resulting of the competitive formation of the (4*S*) enantiomer of the pyrazolidinol **8ab** or **8ac**.

When the asymmetric synthesis of isoxazolidinones **12a**–**12c** was attempted by means of the use of the chiral catalysts **13** and **14** in the reactions between enals **7a**–**7c** and *N*-Cbz-hydroxylamine **10**, the results were much less satisfactory, since after oxidation of the intermediate isoxazolidinols **11a**–**11c** to the corresponding isoxazolidinones **12**, we were not able to find suitable HPLC conditions for the determination of the enantiomeric purity of **12a**; on the other hand, **12b** was obtained in essentially racemic form with both catalysts, and scalemic **12c** could be prepared but only in low enantiomeric purity (57:43 er) when using **14** as the chiral catalyst (see details in the Experimental Section).

### 2.2. Primary Antimicrobial Screening

Finally, and in the framework of the Community for Open Antimicrobial Drug Discovery (CO-ADD) program [31], the new heterocyclic systems synthesized by us (both in racemic and when possible in enantiomerically enriched form) were submitted to a primary antimicrobial screening study by whole cell growth inhibition assays. The inhibition of growth was measured against five bacteria: *Escherichia coli*, *Klebsiella pneumoniae*, *Acinetobacter baumannii*, *Pseudomonas aeruginosa* and *Staphylococcus aureus*, and two fungi: *Candida albicans* and *Cryptococcus neoformans*. The results of the study are summarized in Table 4, which shows the active compounds found for each microbial strain.

Among the samples tested, five showed antimicrobial activity and have been selected for hit confirmation. It is worth noting that antifungal activity was found only for isoxazolidinones (**12a** and **12b**) and for isoxazolidinol **11c**, and that the pyrazolidinol **8aa**, both in racemic and in enantiomerically enriched form, was active against *Staphylococcus aureus*. The fact that neither the corresponding pyrazolidinone **9aa** nor the 4-methyl-1,2-bis-(*p*-toluenesulfonyl)pyrazolidin-3-ol **8ba** show any growth inhibition for the same bacterial strain strongly suggests that 4-methyl-1-(4-nitrobenzenesulfonyl)-4,5-dihydro-1*H*-pyrazole, resulting from acid-catalyzed deprotection/ dehydration of **8aa** [20], could be the biologically active species.

## 3. Experimental Section

### 3.1. General Information

Reactions were generally performed at room temperature, either in round-bottomed flasks or in loosely stoppered glass vials, with magnetic stirring and open to the air. Commercially available reagents, catalysts, and solvents were used as received with the exception of dichloromethane, which was distilled from calcium hydride under nitrogen. Aldehydes **1** [32] and **7b**–**7d** [33,34] were prepared according to literature procedures. Yields refer to products isolated after chromatographic purification. Reactions were monitored both by ^1^H-NMR and by thin-layer chromatography, carried out on silica gel plates Merck 60 F254 (Sigma-Aldrich Química SL, Madrid, Spain), and compounds were visualized by irradiation with UV light and/or treatment with a solution of KMnO_4_ as developing agent followed by heating. Flash column chromatography was performed using silica gel Merck 60 (particle size: 0.040–0.063 mm). ^1^H (400 MHz) and ^13^C (100.6 MHz) NMR spectra were recorded with a Mercury 400 spectrometer (Varian Inc., Palo Alto, CA, USA). Chemical shifts (δ) are given in ppm relative to tetramethylsilane (TMS), and coupling constants (*J*) are given in Hz. The spectra were recorded in CDCl_3_ as solvent at room temperature. TMS served as an internal standard (δ = 0.00 ppm) for ^1^H-NMR spectra, and CDCl_3_ (δ = 77.0 ppm) for ^13^C-NMR spectra. Data are reported as follows: s, singlet; d, doublet; t, triplet; q, quartet; m, multiplet; br, broad signal. Where appropriate, 2D techniques (COSY, NOESY) were also used to assist in structure elucidation. High-resolution mass spectra (HRMS) were recorded with a MicrOTOF spectrometer (Bruker, Billerica, MA, USA) by the Unitat d’Espectrometria de Masses, CCiT-UB. Specific rotations were determined at room temperature with a 241 MC polarimeter (Perkin-Elmer, Waltham, MA, USA). Chiral HPLC analyses were performed with a LC Series 20 apparatus (Shimadzu Corp., Kyoto, Japan) with an M20 diode array UV/Vis detector, using Chiralpak^®^ IC, IA and IB columns (Daicel Corporation, Tokio-Osaka, Japan). The homogeneity of the peaks corresponding to the two enantiomers of the product was thoroughly checked by comparison of the UV spectra.

### 3.2. General Procedures for the Preparation of Pyrazolidines

#### 3.2.1. Racemic Pyrazolidinols from Enals and Hydrazines

An ordinary glass vial equipped with a magnetic stirring bar was charged with pyrrolidine (1.42 mL, 14 mg, 0.20 mmol, 40 mol %), PhCOOH (24 mg, 0.20 mmol, 40 mol %) and toluene (2 mL). The hydrazine **2a** or **2b** (0.50 mmol, 1 eq) and the α,β-unsaturated aldehyde (1.0 mmol, 2 eq) were added sequentially. Stirring was maintained at room temperature until the reaction was complete (TLC and or ^1^H-NMR monitoring, 24 h–4 days) and the crude reaction mixture was diluted with 10 mL of ethyl acetate. The organic phase was washed first with 10% *w*/*w* aqueous sodium bicarbonate solution (10 mL), then with brine (10 mL), and dried over MgSO_4_. Filtration and evaporation of solvents under reduced pressure afforded the crude reaction product that was purified by column chromatography (silica gel; hexane/ethyl acetate mixtures of increasing polarity) to afford the intermediate pyrazolidinol as a diastereomer mixture.

#### 3.2.2. Asymmetric Synthesis of Pyrazolidinols from Enals and Hydrazines

An ordinary glass vial equipped with a magnetic stirring bar was charged with the chiral prolinol silyl ether **13** or **14** (0.05 mmol, 20 mol %), PhCOOH (6 mg, 0.05 mmol, 20 mol %) and toluene (1 mL). The hydrazine **2a** or **2b** (0.24 mmol, 1 eq) and the α,β-unsaturated aldehyde (0.48 mmol, 2 eq) were added sequentially. The stirring was maintained at room temperature until the reaction was complete (TLC and or ^1^H-NMR monitoring, 3–6 days) and the crude reaction mixture was diluted with 10 mL of ethyl acetate. The organic phase was washed first with 10% *w*/*w* aqueous sodium bicarbonate solution (10 mL), then with brine (10 mL), and dried over MgSO_4_. Filtration and evaporation of solvents under reduced pressure afforded the crude reaction product that was purified by column chromatography (silica gel; hexane/ethyl acetate mixtures of increasing polarity) to afford the intermediate pyrazolidinol as a diastereomer mixture.

#### 3.2.3. Pyrazolidinones by Oxidation of Pyrazolidinols

The pyrazolidinol diastereomer mixture (0.15 mmol) and pyridinium chlorochromate PCC (158 mg, 0.73 mmol, 5 eq) were added sequentially to a stirred suspension of activated 4 Å molecular sieves (300 mg) in anhydrous dichloromethane (2 mL), and the reaction mixture was stirred at room temperature until completion (24–48 h). After the addition of diethyl ether (10 mL) to precipitate the chromium salts, the solution was decanted and filtered through a short pad of Celite^®^ eluting with a 1:10 mixture of ethyl acetate and diethyl ether). After removal of the solvents in vacuo, the reaction product was purified by column chromatography (silica gel, hexane/ethyl acetate mixtures) to give the desired pyrazolidinone.

### 3.3. General Procedures for the Preparation of Isoxazolidines

#### 3.3.1. Racemic Isoxazolidinols from Enals and Hydroxylamine **10**

Pyrrolidine (from a 10 mg/mL solution in toluene; 1.48 mL, 0.21 mmol, 40 mol %) and *N*-Cbz-hydroxylamine **10** (87 mg, 0.52 mmol, 1 eq) were added sequentially to a magnetically stirred solution of the α,β-unsaturated aldehyde **7a**, **7b**, or **7c** (1.04 mmol, 2 eq) in toluene (2 mL), and the resulting solution was stirred at room temperature. The progress of the reaction was monitored both by ^1^H-NMR spectroscopy and by TLC. When the starting hydroxylamine **10** was not detected (2 days), toluene and pyrrolidine were removed in vacuo, and the crude residue was directly purified by column chromatography (silica gel; hexane/ethyl acetate mixtures) to give the corresponding isoxazolidinol as a diastereomer mixture.

#### 3.3.2. Asymmetric Synthesis of Isoxazolidinols from Enals and Hydroxylamine **10**

The chiral prolinol silyl ether **13** or **14** (0.104 mmol, 20 mol %) and *N*-Cbz-hydroxylamine **10** (87 mg, 0.52 mmol, 1 eq) were added sequentially to a magnetically stirred solution of the α,β-unsaturated aldehyde **7a**, **7b**, or **7c** (1.04 mmol, 2 eq) in toluene (2 mL), and the resulting solution was stirred at room temperature. The progress of the reaction was monitored both by ^1^H-NMR spectroscopy and by TLC. When the starting hydroxylamine **10** was not detected (3 days), toluene was removed in vacuo, and the crude residue was directly purified by column chromatography (silica gel; hexane/ethyl acetate mixtures) to give the corresponding isoxazolidinol as a diastereomer mixture.

#### 3.3.3. Isoxazolidinones by Oxidation of Isoxazolidinols

The isoxazolidinol diastereomer mixture (0.126 mmol) and pyridinium chlorochromate PCC (82 mg, 0.38 mmol, 5 eq) were added sequentially to a stirred suspension of activated 4Å molecular sieves (150 mg) in anhydrous dichloromethane (2 mL), and the reaction mixture was stirred overnight at room temperature. After the addition of diethyl ether (5 mL) to precipitate the chromium salts, the solution was decanted and filtered through a short pad of Celite^®^ eluting with a 4:1 mixture of hexane and ethyl acetate. After removal of the solvents in vacuo, the reaction product was purified by column chromatography (silica gel, hexane/ethyl acetate mixtures) to give the desired isoxazolidinone.

### 3.4. Characterization of the Products

*tert-Butyl (3RS,3aRS,6aSR)-3-hydroxy-1-((4-nitrophenyl)sulfonyl)hexahydrocyclopenta[c]pyrazole-2(1H)-carboxylate*, **3**. Obtained in 87% yield (450 mg, 1.09 mmol) according to Procedure 3.2.1. Colorless solid, m.p. 83–85 °C (from 1:9 chloroform/hexanes). ^1^H-NMR (CDCl_3_): δ = 1.24 (s, 9H), 1.47–1.54 (m, 3H), 1.67–1.75 (m, 1H), 1.77–1.83 (m, 1H), 1.86–1.95 (m, 1H), 2.72–2.77 (m, 1H), 3.38 (br d, 1H, OH), 4.73–4.78 (m, 1H), 5.42 (m, 1H), 8.11 (d, *J* = 8.0 Hz, 2H), 8.29 (d, *J* = 8.0 Hz, 2H) ppm. ^13^C-NMR (CDCl_3_): δ = 24.5, 27.8, 30.5, 33.7, 53.6, 62.2, 65.5, 83.0, 91.9, 123.7, 131.1, 141.8, 150.7 ppm. HRMS (ESI): Calculated for C_17_H_27_N_4_O_7_S [M + NH_4_]^+^ = 431.1595; found, 431.1592.

*tert-Butyl 3,4-trans-5-hydroxy-3,4-dimethyl-2-((4-nitrophenyl)sulfonyl)-pyrazolidine-1-carboxylate*, **5**. Obtained as a 6:1 diastereomer mixture in 76% yield (380 mg, 0.95 mmol) according to Procedure 3.2.1. Colorless solid, m.p. 71–73 °C (major isomer, 1:9 chloroform/hexanes). ^1^H-NMR (CDCl_3_): δ = 1.00 (d, *J* = 7.0 Hz, 3H), 1.24 (s, 9H), 1.36 (d, *J* = 7.0 Hz, 3H), 1.80–1.87 (m, 1H), 2.86 (br d, 1H, OH), 3.72–3.79 (m, 1H), 5.46–5.48 (m, 1H), 8.07 (d, *J* = 8.0 Hz, 2H), 8.28 (d, *J* = 8.0 Hz, 2H) ppm. ^13^C-NMR (CDCl_3_): δ = 11.1, 20.7, 27.81, 27.83, 47.4, 62.2, 83.2, 86.6, 123.6, 130.9, 141.8, 150.6, 154.7 ppm. HRMS (ESI): Calculated for C_16_H_24_N_3_O_7_S [M + H]^+^ = 402.1337; found, 402.1340.

*tert-Butyl 5-hydroxy-4-methyl-2-((4-nitrophenyl)sulfonyl)-pyrazolidine-1-carboxylate*, **8aa**. The racemic compound was obtained as a 4:1 diastereomer mixture in 93% yield (180 mg, 0.47 mmol) according to Procedure 3.2.1. Colorless solid, m.p. 111–114 °C (major isomer, 1:9 chloroform/hexanes). ^1^H-NMR (CDCl_3_): δ = 1.09 (d, *J* = 7.0 Hz, 3H), 1.35 (s, 9H), 2.25–2.30 (m, 1H), 3.03–3.09 (m, 1H), 3.22 (br d, 1H, OH), 3.29–3.36 (m, 1H), 3.83–3.87 (m, 1H), 5.47 (m, 1H), 8.12 (d, *J* = 8.0 Hz, 2H), 8.34 (d, *J* = 8.0 Hz, 2H) ppm. ^13^C-NMR (CDCl_3_): δ = 15.0, 27.9, 43.0, 55.3, 83.2, 91.9, 123.9, 131.0, 141.8, 150.8, 154.5 ppm. HRMS (ESI): Calculated for C_15_H_21_N_3_NaO_7_S [M + Na]^+^ = 410.0992; found, 410.1000. When using **14** as a catalyst and following Procedure 3.2.2, scalemic **8aa** was obtained in 98.6% yield (3:1 diastereomer mixture). Colorless solid, m.p. = 135–137 °C (major isomer, 1:9 chloroform/hexanes).

*(−)-tert-Butyl 4-methyl-2-((4-nitrophenyl)sulfonyl)-pyrazolidin-5-one-1-carboxylate*, **9aa**. Oxidation of **8aa** obtained by catalyst **14** gave (−)-**9aa** (90:10 er) in 94% yield (101 mg, 0.26 mmol) according to Procedure 3.2.3. Colorless solid, m.p. 131–134 °C (from 1:9 chloroform/hexanes). ^1^H-NMR (CDCl_3_): δ = 1.16 (d, *J* = 7.0 Hz, 3H), 1.47 (s, 9H), 2.54–2.64 (m, 1H), 3.38–3.45 (m, 1H), 4.40–4.45 (m, 1H), 8.18 (d, *J* = 8.0 Hz, 2H), 8.41 (d, *J* = 8.0 Hz, 2H) ppm. ^13^C-NMR (CDCl_3_): δ = 13.4, 27.7, 36.8, 53.7, 85.6, 124.4, 130.7, 141.3, 151.4, 173.4 ppm. HRMS (ESI): Calculated for C_15_H_19_N_3_NaO_7_S [M + Na]^+^ = 408.0836; found, 408.0847. [α]${}_{D}^{25}$ = −56.0 (1.23, DCM). Conditions for the HPLC analysis: Chiralpak^®^ IC column, 90:10 hexane/2-propanol, 1 μL/min, 25 °C, λ = 254 nm. t_R_ (major enant.) = 58.1 min, t_R_ (minor enant.) = 84.4 min.

*tert-Butyl 4-benzyl-5-hydroxy-2-((4-nitrophenyl)sulfonyl)-pyrazolidine-1-carboxylate*, **8ab**. The racemic compound was obtained in 83% yield (165 mg, 0.36 mmol, >20:1 dr) according to procedure 3.2.1. Colorless solid, m.p. 122–126 °C (major isomer, recrystallized from 1:2:7 dichloromethane/chloroform/hexanes). ^1^H-NMR (CDCl_3_): δ = 1.36 (s, 9H), 2.53–2.58 (m, 2H), 3.14–3.19 (m, 1H), 3.24 (br d, 1H, OH), 4.01–4.06 (m, 1H), 5.39–5.41 (m, 1H), 7.09 (d, *J* = 7.8 Hz, 2H), 7.20–7.33 (m, 3H), 8.13 (d, *J* = 8.0 Hz, 2H), 8.34 (d, *J* = 8.0 Hz, 2H) ppm. ^13^C-NMR (CDCl_3_): δ = 27.9, 36.7, 50.1, 53.7, 83.3, 89.9, 123.8, 126.9, 128.4, 128.8, 131.0, 137.8, 141.7, 150.8, 154.3 ppm. HRMS (ESI): Calculated for C_21_H_25_N_3_NaO_7_S [M + Na]^+^ = 486.1305; found, 486.1298. When using **13** as a catalyst and following Procedure 3.2.2, scalemic **8ab** was obtained in 77% yield. Colorless solid, m.p. = 132–136 °C (major isomer, 1:2:7 dichloromethane/chloroform/hexanes).

*(−)-tert-Butyl 4-benzyl-2-((4-nitrophenyl)sulfonyl)-pyrazolidin-5-one-1-carboxylate*, **9ab**. Oxidation of **8ab** obtained by catalyst **13** gave (−)-**9ab** (44:56 er) in 98% yield (108 mg, 0.23 mmol) according to Procedure 3.2.3. Colorless solid, m.p. 115–118 °C (recrystallized from 1:2:7 dichloromethane/chloroform/hexanes). ^1^H-NMR (CDCl_3_): δ = 1.46 (s, 9H), 2.61–2.67 (m, 1H), 2.86–2.92 (m, 1H), 3.17–3.21 (m, 1H), 3.46–3.52 (m, 1H), 4.16–4.21 (m, 1H), 7.07 (d, *J* = 6.7 Hz, 2H), 7.25–7.32 (m, 3H), 8.13 (d, *J* = 7.8 Hz, 2H), 8.37 (d, *J* = 7.8 Hz, 2H) ppm. ^13^C-NMR (CDCl_3_): δ = 27.7, 34.8, 43.6, 51.7, 85.7, 124.3, 127.2, 128.5, 130.7, 136.7, 141.2, 147.5, 151.4, 172.1 ppm. HRMS (ESI): Calculated for C_42_H_50_N_7_O_14_S_2_ [2M + NH4]^+^ = 940.2852; found, 940.2847. [α]${}_{D}^{25}$ = −11.4 (1.19, DCM). Conditions for the HPLC analysis: Chiralpak^®^ IC column, 90:10 hexane/2-propanol, 1 μL/min, 25 °C, λ = 254 nm. t_R_ (minor enant.) = 62.4 min, t_R_ (major enant.) = 73.2 min.

*tert-Butyl 4-hexyl-5-hydroxy-2-((4-nitrophenyl)sulfonyl)-pyrazolidine-1-carboxylate*, **8ac**. Obtained in 99.6% yield as a diastereoisomer mixture (227 mg, 1.00 mmol, 5:1 dr) according to Procedure 3.2.1. Colorless solid, m.p. 83–85 °C. ^1^H-NMR (CDCl_3_, major isomer): δ = 0.86 (t, *J* = 7Hz, 3H), 1.25–1.32 (m, 8H), 1.33 (s, 9H), 1.49–1.56 (m, 2H), 2.15–2.19 (m, 1H), 3.01–3.07 (m, 1H), 3.25 (br d, 1H, OH), 4.19–4.24 (m, 1H), 5.25–5.28 (m, 1H), 8.17 (d, *J* = 8.0 Hz, 2H), 8.36 (d, *J* = 8.0 Hz, 2H) ppm. HRMS (ESI): Calculated for C_40_H_62_N_6_NaO_14_S_2_ [2M + Na]^+^ = 937.3658; found, 937.3654.

*tert-Butyl 4-hexyl-2-((4-nitrophenyl)sulfonyl)-pyrazolidin-5-one-1-carboxylate*, **9ac**. Obtained in 99% yield (150 mg, 0.33 mmol) according to Procedure 3.2.3. Colorless solid, m.p. 123–126 °C. ^1^H-NMR (CDCl_3_): δ = 0.87 (t, *J* = 6.5 Hz, 3H), 1.24–1.30 (m, 9H), 1.47 (s, 9H), 1.78–1.83 (m, 1H), 2.40–2.47 (m, 1H), 3.42–3.49 (m, 1H), 4.37–4.42 (m, 1H), 8.19 (d, *J* = 7.8 Hz, 2H), 8.42 (d, *J* = 7.8 Hz, 2H) ppm. ^13^C-NMR (CDCl_3_): δ = 13.9, 22.5, 26.8, 27.7, 28.8, 29.1, 31.4, 41.8, 52.6, 124.4, 130.7, 141.3, 147.7, 151.3, 172.9 ppm. HRMS (ESI): Calculated for C_20_H_29_N_3_NaO_7_S [M + Na]^+^ = 478.1618; found, 478.1627.

*4-Methyl-1,2-bis-(4-toluenesulfonyl)-pyrazolidin-3-ol*, **8ba**. The racemic compound was obtained as a 8:1 diastereomer mixture in 88% yield (75 mg, 0.18 mmol) according to Procedure 3.2.1. Yellow solid, m.p. 131–134 °C. ^1^H-NMR (CDCl_3_, major isomer): δ = 0.86 (d, *J* = 7.0 Hz, 3H), 2.17–2.25 (m, 2H), 2.45 (s, 3H), 2.46 (s, 3H), 3.33–3.40 (m, 1H), 4.04–4.11 (m, 1H), 5.21–5.22 (m, 1H), 7.29–7.33 (m, 4H), 7.70 (d, *J* = 8.0 Hz, 2 H), 7.82 (d, *J* = 8.0 Hz, 2 H) ppm. HRMS (ESI): Calculated for C_36_H_44_N_4_NaO_10_S_4_ [2M + Na]^+^ = 843.1832; found, 843.1832. When using **13** as a catalyst and following Procedure 3.2.2, scalemic **8ba** was obtained in 89.4% yield (8:1 diastereomer mixture).

*(−)-4-Methyl-1,2-bis-(4-toluenesulfonyl)-pyrazolidin-3-one*, **9ba**. Oxidation of **8ba** obtained by catalyst **13** gave (−)-**9ba** (96:4 er) in 98% yield (58 mg, 0.14 mmol) according to Procedure 3.2.3. Colorless solid, m.p. 156–160 °C (recrystallized from 1:2:7 dichloromethane/chloroform/hexanes). ^1^H-NMR (CDCl_3_): δ = 0.76 (d, *J* = 7.0 Hz, 3H), 2.12–2.23 (m, 1H), 2.46 (s, 6H), 2.83–2.90 (m, 1H), 4.28–4.33 (m, 1H), 7.34–7.36 (m, 4H), 7.89 (d, *J* = 8.1 Hz, 2H), 7.89 (d, *J* = 8.1 Hz, 2H) ppm. ^13^C-NMR (CDCl_3_): δ = 15.3, 21.8, 34.7, 65.8, 127.0, 128.3, 128.7, 128.8, 129.6, 129.8, 130.1, 146.0, 146.2, 176.5 ppm. HRMS (ESI): Calculated for C_36_H_40_N_4_NaO_10_S_2_ [2M + Na]^+^ = 839.1519; found, 839.1516. [α]${}_{D}^{25}$ = −64.7 (0.67, DCM). Conditions for the HPLC analysis: Chiralpak^®^ IA column, 90:10 hexane/2-propanol, 1 μL/min, 25 °C, λ = 254 nm. t_R_ (major enant.) = 33.6 min, t_R_ (minor enant.) = 51.5 min.

*4-Benzyl-1,2-bis-(4-toluenesulfonyl)-pyrazolidin-3-ol*, **8bb**. The racemic compound was obtained as a diastereomer mixture (>9:1 dr) in 85% yield (85 mg, 0.18 mmol) according to Procedure 3.2.1. Yellow solid, m.p. 110–113 °C. ^1^H-NMR (CDCl_3_, major isomer): δ = 2.14–2.20 (m, 1H), 2.35–2.42 (m, 2H), 2.46 (s, 3H), 2.47 (s, 3H), 2.82–2.87 (m, 1H), 3.45–3.47 (br d, 1H, OH), 3.89–3.94 (m, 1H), 5.36–5.40 (m, 1H), 7.28–7.31 (m, 5H), 7.67 (d, *J* = 8.0 Hz, 2H), 7.77 (d, *J* = 8.0 Hz, 2H) ppm. HRMS (ESI): Calculated for C_48_H_56_N_5_O_10_S_4_ [2M + NH_4_]^+^ = 990.2805; found, 990.2805. When using **13** or **14** as catalysts and following Procedure 3.2.2, scalemic **8bb** was obtained in the same yield and diastereomeric ratio.

*4-Benzyl-1,2-bis-(4-toluenesulfonyl)-pyrazolidin-3-one*, **9bb**. Oxidation of **8bb** according to Procedure 3.2.3 gave **9bb** in 97% yield (58 mg, 0.12 mmol). Yellow solid, m.p. 156–160 °C. ^1^H-NMR (CDCl_3_): δ = 2.01–2.07 (m, 1H), 2.45–2.47 (m, 1H), 2.47 (s, 3H), 2.48 (s, 3H), 2.90–2.95 (m, 1H), 2.98–3.01 (m, 1H), 4.05–4.011 (m, 1H), 6.81 (m, 1H), 7.20 (m, 3H), 7.34 (d, *J* = 6 Hz, 4H), 7.73 (d, *J* = 7.6 Hz, 2H), 7.86 (d, *J* = 7.6 Hz, 2H) ppm. ^13^C-NMR (CDCl_3_): δ = 21.79, 21.82, 34.7, 42.7, 54.0, 127.0, 128.3, 128.7, 128.8, 129.6, 129.8, 130.1, 131.2, 134.67, 134.73, 146.0, 146.2, 176.5 ppm. HRMS (ESI): Calculated for C_24_H_28_N_3_NaO_5_S_2_ [M + NH_4_]^+^ = 502.1465; found, 502.1466. No satisfactory conditions for the chiral HPLC analysis of this compound could be found.

*4-Hexyl-1,2-bis-(4-toluenesulfonyl)-pyrazolidin-3-ol*, **8bc**. The racemic compound was obtained as a diastereomer mixture (10:1 dr) in 98% yield (97 mg, 0.20 mmol) according to Procedure 3.2.1. Yellow solid, m.p. 78–80 °C. ^1^H-NMR (CDCl_3_, major isomer): δ = 0.87 (t, *J* = 7.0 Hz, 3H), 0.95–1.00 (m, 1H), 1.13–1.18 (m, 6H), 1.22–1.27 (m, 3H), 2.01–2.14 (m, 1H), 2.44 (s, 3H), 2.45 (s, 3H), 2.85–2.87 (m, 1H), 3.35–3.37 (br d, 1H, OH), 4.08–4.13 (m, 1H), 5.24–5.28 (m, 1H), 7.27–7.32 (m, 4H), 7.68 (d, *J* = 8.0 Hz, 2H), 7.81 (d, *J* = 8.0 Hz, 2H) ppm. HRMS (ESI): Calculated for C_23_H_32_N_2_NaO_5_S_2_ [M + Na]^+^ = 503.1642; found, 503.1645. When using **13** as a catalyst and following Procedure 3.2.2, scalemic **8bc** was obtained in 93% yield (104 mg, 0.23 mmol; 10:1 dr).

*(−)-4-Hexyl-1,2-bis-(4-toluenesulfonyl)-pyrazolidin-3-one*, **9bc**. Oxidation of **8bc** (obtained by catalyst **13**) according to Procedure 3.2.3 gave (−)-**9bc** (78:22 er) in 98% yield (58 mg, 0.12 mmol). Colourless solid, m.p. 160–164 °C. ^1^H-NMR (CDCl_3_): δ = 0.83–0.86 (m, 5H), 1.05–1.12 (m, 4H), 1.19–1.25 (m, 3H), 1.40–1.52 (m, 1H), 2.08–2.11 (m, 1H), 2.45 (s, 3H), 2.46 (s, 3H), 2.93–3.00 (m, 1H), 4.24–4.29 (m, 1H), 7.33–7.36 (m, 4H), 7.78 (d, *J* = 8.0 Hz, 2H), 7.88 (d, *J* = 8.0 Hz, 2H) ppm. ^13^C-NMR (CDCl_3_): δ = 13.9, 21.7, 21.8, 22.4, 26.2, 28.6, 28.7, 31.3, 41.0, 54.1, 128.7, 129.6, 129.8, 130.1, 131.3, 134.8, 145.9, 146.2, 177.5 ppm. HRMS (ESI): Calculated for C_23_H_30_N_2_NaO_5_S_2_ [M + Na]^+^ = 501.1488; found, 501.1491. [α]${}_{D}^{25}$ = −8.0 (1.01, DCM). Conditions for the HPLC analysis: Chiralpak^®^ IB column, 93:7 hexane/2-propanol, 1 μL/min, 25 °C, λ = 220 nm. t_R_ (major enant.) = 21.5 min, t_R_ (minor enant.) = 27.6 min.

*Benzyl 5-hydroxy-4-methyl-isoxazolidine-2-carboxylate*, **11a**. The racemic compound was obtained as a 2:1 diastereomer mixture in 83% yield (102 mg, 0.43 mmol) according to Procedure 3.3.1. Colorless oil. ^1^H-NMR (CDCl_3_, major isomer): δ = 1.09 (d, *J* = 7 Hz, 3H), 2.60–2.67 (m, 1H), 3.61–3.64 (m, 1H), 4.26–4.29 (m, 1H), 5.16 (br s, 2H), 5.34–5.35 (m, 1H), 7.32–7.35 (m, 5H) ppm. ^1^H-NMR (CDCl_3_, minor isomer): δ = 1.13 (d, *J* = 7.0 Hz, 3H), 2.55–2.59 (m, 1H), 3.78–3.82 (m, 1H), 4.14–4.17 (m, 1H), 5.22 (br s, 2H), 5.55–5.56 (m, 1H), 7.37–7.39 (m, 5H) ppm. ^13^C-NMR (CDCl_3_): δ = 9.4, 14.7, 40.4, 44.1, 67.8, 68.1, 73.5, 74.9, 82.1, 88.0, 128.29, 128.34, 128.43, 128.46, 128.5, 128.6, 135.38, 135.44, 141.8, 155.3, 159.0 ppm. When using **13** or **14** as catalysts and following Procedure 3.3.2, scalemic **11a** was obtained in similar yield and diastereomer ratio.

*Benzyl 4-methyl-5-oxo-isoxazolidine-2-carboxylate*, **12a**. Oxidation of **11a** gave **12a** in 98% yield (50 mg, 0.22 mmol) according to Procedure 3.3.3. Colorless oil. ^1^H-NMR (CDCl_3_): δ = 1.29 (d, *J* = 6.8 Hz, 3H), 3.01–3.11 (m, 1H), 3.96–4.01 (m, 1H), 4.54–4.58 (m, 1H), 5.33 (br s, 2H), 7.33–7.45 (m, 5H) ppm.^13^C-NMR (CDCl_3_): δ = 12.2, 39.4, 68.8, 73.9, 98.0, 128.54, 128.6, 134.5, 147.7, 169.9 ppm. HRMS (ESI): Calculated for C_12_H_14_NO_4_ [M + H]^+^ = 236.0917; found, 236.0916. No satisfactory conditions could be found for the chiral HPLC analysis of this compound.

*Benzyl 4-benzyl-5-hydroxy-isoxazolidine-2-carboxylate*, **11b**. The racemic compound was obtained as a 3:1 diastereomer mixture in 79% yield (128 mg, 0.41 mmol) according to Procedure 3.3.1. Colorless oil. ^1^H-NMR (CDCl_3_, major isomer): δ = 2.74–2.78 (m, 2H), 3.02–3.06 (m, 1H), 3.57–3.61 (m, 1H), 3.77–3.80 (m, 1H), 4.19–4.23 (m, 1H), 5.25 (br s, 2H), 5.47–5.48 (m, 1H), 7.31–7.41 (m, 10H) ppm. ^1^H-NMR (CDCl_3_, minor isomer): δ = 2.59–2.68 (m, 2H), 2.94–2.98 (m, 1H), 3.01–3.27 (m, 1H), 3.67–3.71 (m, 1H), 4.01–4.05 (m, 1H), 5.36 (br s, 2H), 5.56–5.57 (m, 1H), 7.13–7.30 (m, 10H) ppm. ^13^C-NMR (CDCl_3_): δ = 23.5, 31.2, 35.8, 48.0, 48.8, 51.0, 68.1, 71.9, 72.9, 81.3, 86.1, 97.2, 101.4, 126.5, 126.7, 128.1, 128.2, 128.4, 128.5, 128.6, 128.7, 135.5, 135.7, 138.2, 138.9, 155.2, 158.8 ppm.

*Benzyl 4-benzyl-5-oxo-isoxazolidine-2-carboxylate*, **12b**. Oxidation of **11b** gave **12b** in 99% yield (68 mg, 0.22 mmol) according to Procedure 3.3.3. Colorless oil. ^1^H-NMR (CDCl_3_): δ = 2.78–2.84 (m, 1H), 3.24–3.28 (m, 2H), 4.07–4.11 (m, 1H), 4.35–4.39 (m, 1H), 5.33 (br s, 2H), 7.15–7.43 (m, 10H) ppm.^13^C-NMR (CDCl_3_): δ = 30.7, 43.0, 65.7, 68.8, 123.9, 125.3, 125.4, 125.5, 125.7, 131.3, 134.0, 144.4, 165.4 ppm. HRMS (ESI): Calculated for C_18_H_21_N_2_O_4_ [M + NH_4_]^+^ = 329.1496; found, 329.1494.

*Benzyl 4-hexyl-5-hydroxy-isoxazolidine-2-carboxylate*, **11c**. The racemic compound was obtained as a 3:1 diastereomer mixture in 78% yield (120 mg, 0.39 mmol) according to Procedure 3.3.1. Colorless oil. ^1^H-NMR (CDCl_3_, major isomer): δ = 0.88 (d, *J* = 7.0 Hz, 3H), 1.28–1.31 (m, 9H), 1.34–1.38 (m, 1H), 2.45–2.55 (m, 2H), 3.62–3.66 (m, 1H), 4.26–4.28 (m, 1H), 5.22 (br s, 2H), 5.39–5.40 (m, 1H), 7.37–7.40 (m, 5H) ppm. ^1^H-NMR (CDCl_3_, minor isomer): δ = 0.87 (d, *J* = 7.0 Hz, 3H), 1.34–1.38 (m, 9H), 3.15–3.19 (m, 2H), 3.81–3.86 (m, 1H), 4.02–4.25 (m, 1H), 5.33 (br s, 2H), 5.39–5.40 (m, 1H), 7.32–7.36 (m, 5H) ppm. When using **14** as a catalyst and following Procedure 3.3.2, scalemic **11c** was obtained in 87% yield (201 mg, 0.65 mmol) and in a 3:1 diastereomer ratio.

*(−)-Benzyl 4-hexyl-5-oxo-isoxazolidine-2-carboxylate*, **12c**. Oxidation of **11c** obtained with catalyst **14** gave (−)-**12c** (57:43 er) in 87% yield (87 mg, 0.28 mmol) according to Procedure 3.3.3. Colorless oil. ^1^H-NMR (CDCl_3_): δ = 0.88 (d, *J* = 7.0 Hz, 3H), 1.28–1.40 (m, 8H), 1.50–1.58 (m, 1H), 1.88–1.93 (m, 1H), 2.92–2.99 (m, 1H), 4.04–4.08 (m, 1H), 4.52–4.56 (m, 1H), 5.33 (br s, 2H), 7.33–7.45 (m, 5H) ppm. ^13^C-NMR (CDCl_3_): δ = 14.0, 22.5, 27.0, 28.1, 29.0, 31.5, 44.5, 68.8, 72.6, 128.5, 128.6, 128.7, 134.6, 147.7, 169.5 ppm. HRMS (ESI): Calculated for C_34_H_46_N_2_NaO_8_ [2M + Na]^+^ = 633.3146; found, 633.3148. [α]${}_{D}^{25}$ = −4.1 (0.41, DCM). Conditions for the HPLC analysis: Chiralpak^®^ IB column, 90:10 hexane/2-propanol, 1 μL/min, 25 °C, λ = 254 nm. t_R_ (minor enant.) = 13.6 min, t_R_ (major enant.) = 16.3 min.

### 3.5. Methods for the Biological Activity Studies

#### 3.5.1. Sample Preparation

Samples were provided as dry material, and were made to 10 mg/mL in DMSO solution and stored at −20 °C. An aliquot of each sample was diluted to 320 μg/mL in water, and plated in 384-well polypropylene plates. 5 μL was plated in duplicate into a 384-well non-binding surface plate for each strain or cell type assayed against. Once cells were added this gave a final compound concentration range of 32 μg/mL. The final DMSO concentration was 0.3%.

#### 3.5.2. Antimicrobial Assay

##### Procedure

All bacteria were cultured in Cation-adjusted Müller Hinton broth (CAMBH) at 37 °C overnight. A sample of each culture was then diluted 40-fold in fresh broth and incubated at 37 °C for 1.5–3 h. The resulting mid-log phase cultures were diluted (CFU/mL measured by OD_600_), and then 45 μL was added to each well of the compound containing plates, giving a cell density of 5 × 10^5^ CFU/mL and the nominated final compound concentration. All the plates were covered and incubated at 37 °C for 18 h without shaking.

##### Analysis

Inhibition of bacterial growth was determined measuring absorbance at 600 nm (OD_600_), using a M1000 Pro monochromator plate reader (Tecan, Männedorf, Switzerland). The percentage of growth inhibition was calculated for each well, using negative control (media only) and positive control (bacteria without inhibitors) on the same plate as references. The significance of the inhibition values was determined by Z-scores, calculated using the average and standard deviation of the sample wells (no controls) on the same plate. Samples with inhibition value above 80% and Z-Score above 2.5 (*n* = 2 on different plates) for either replicate were classed as actives.

#### 3.5.3. Antifungal Assay

##### Procedure

Fungi strains were cultured for 3 days on Yeast Extract-Peptone Dextrose (YPD) agar at 30 °C. A yeast suspension of 1 × 10^6^ to 5 × 10^6^ cells/mL (as determined by OD_350_) was prepared from five colonies. These stock suspensions were diluted with Yeast Nitrogen Base (YNB) broth to a final concentration of 2.5 × 10^3^ CFU/mL. Then, 45 μL of the fungi suspension was added to each well of the compound-containing plates, giving a final concentration of 32 μg/mL for the tested samples. Plates were covered and incubated at 35 °C for 24 h without shaking.

##### Analysis

Growth inhibition of *C. albicans* was determined measuring absorbance at 530 nm (OD_530_), while the growth inhibition of *C. neoformans* was determined measuring the difference in absorbance between 600 and 570 nm (OD_600–570_), after the addition of resazurin (0.001% final concentration) and incubation at 35 °C for additional 2 h. The absorbance was measured using a Biotek Synergy HTX plate reader. The percentage of growth inhibition was calculated for each well, using negative control (media only) and positive control (fungi without inhibitors) on the same plate as references. The significance of the inhibition values was determined by Z-scores, calculated using the average and standard deviation of the sample wells (no controls) on the same plate. Samples with inhibition value above 80% and Z-Score above 2.5 (*n* = 2 on different plates) for either replicate were classed as actives.

#### 3.5.4. Antibiotic Standards Preparation and Quality Control

Colistin and vancomycin were used as positive bacterial inhibitor standards for Gram-negative and Gram-positive bacteria, respectively. Fluconazole was used as a positive fungal inhibitor standard both for *C. albicans* and *C. neoformans*.

The antibiotics were provided in four concentrations, with two above and two below its MIC value, and plated into the first 8 wells of column 23 of the 384-well NBS plates. The quality control (QC) of the assays was determined by the antimicrobial controls and the Z’-factor (using positive and negative controls). Each plate was deemed to fulfill the quality criteria (pass QC), if the Z’-factor was above 0.4, and the antimicrobial standards showed full range of activity, with full growth inhibition at their highest concentration, and no growth inhibition at their lowest concentration.

#### 3.5.5. Materials

Both the compound preparation plate and the assay plates were purchased from Corning (Corning, NY, USA). CAMHB from Bacto Laboratories (Mount Pritchard, Australia) was used as growth media for bacteria; culture agar and growth media for fungi were purchased from Becton Dickinson (Franklin Lakes, NJ, USA). Resazurin was provided by Sigma Aldrich (Sydney, Australia).

#### 3.5.6. Microbial Strains


*Escherichia coli*ATCC 25922 (FDA control strain)*Klebsiella pneumoniae*ATCC 700603 (MDR)*Acinetobacter baumannii*ATCC 19606 (type strain)*Pseudomonas aeruginosa*ATCC 27853 (Quality control strain)*Staphylococcus aureus*ATCC 43300 (MRSA)*Candida albicans*ATCC 90028 (CLSI reference)*Cryptococcus neoformans*ATCC 208821 (H99—Type strain)


## 4. Conclusions

The pyrrolidine-catalyzed reaction of the α-substituted enals methacrolein (**7a**), 2-benzylpropenal (**7b**), and 2-(*n*-hexyl)propenal (**7c**) with the activated hydrazines 1-Boc-2-(4-nitrobenzenesulfonyl)hydrazine (**2a**) and 1,2-bis(*p*-toluenesulfonyl)hydrazine (**2b**) takes place in very good yields (83%–99.6%) in toluene at room temperature and in the presence of benzoic acid as a co-catalyst, to afford the 4-substituted pyrazolidin-3-ols **8aa**–**8bc** (generally as diastereomer mixtures); oxidation of these compounds with PCC leads to the corresponding-4-substituted-3-pyrazolidinones **9aa**–**9bc** in essentially quantitative yields. In the same conditions α-substituted acroleins **7a**–**7c** react smoothly with *N*-Cbz-hydroxylamine **10** to afford the isoxazolidinols **11** in good yields (78%–83% isolated yields) as diastereomer mixtures. Subsequent oxidation with PCC gives rise to the 4-substituted isoxazolidin-5-ones **12a**–**12c**. The use of chiral diarylprolinol trimethylsilyl ethers **13** and **14** as catalysts allows the synthesis of several of these compounds in optically active form, in some cases with excellent enantioselectivity (up to 96:4 er). The 4-substituted pyrazolidinols, pyrazolidinones, isoxazolidinols and isoxazolidinones (both in racemic and when possible in enantiomerically enriched form) have been submitted to a primary antimicrobial screening study (antibacterial and antifungal) by whole cell growth inhibition assays. Antifungal activity has been found for the isoxazolidinones **12a** and **12b**, and for isoxazolidinol **11c**, and the 4-methyl-pyrazolidinol **8aa**, both in racemic and in enantiomerically enriched form, is highly active against *Staphylococcus aureus*.

## Figures and Tables

**Table 1:** Pyrrolidine-catalyzed synthesis of pyrazolidin-3-ols **8aa**–**8bc** from α-substituted enals **7a**–**7c** and hydrazines **2a** and **2b**, and oxidation to the corresponding pyrazolidin-3-ones **9aa**–**9bc**.

Entry	Reactants	R	X	Y	Pyrazolidinol	Yield ^1^	Reaction Time (Day)	dr ^2^	Pyrazolidinone	Yield ^1^
1	**7a, 2a**	Me	Ns	Boc	**8aa**	93%	4	4:1	**9aa**	94%
2	**7b, 2a**	PhCH_2_	Ns	Boc	**8ab**	83%	2	>20:1	**9ab**	98%
3	**7c, 2a**	*n*-Hexyl	Ns	Boc	**8ac**	99.6%	2	5:1	**9ac**	99%
4	**7a, 2b**	Me	Ts	Ts	**8ba**	88%	2	8:1	**9ba**	98%
5	**7b, 2b**	PhCH_2_	Ts	Ts	**8bb**	85%	2	9:1	**9bb**	97%
6	**7c, 2b**	*n*-Hexyl	Ts	Ts	**8bc**	98%	2	10:1	**9bc**	98%

^1^ Yield of isolated product after chromatographic purification; ^2^ By ^1^H-NMR.

**Table 2:** Pyrrolidine-catalyzed synthesis of isoxazolidin-5-ols **11a**–**11c** from α-substituted enals **7a**–**7c** and *N*-Cbz-hydroxylamine **10**, and oxidation to the corresponding isoxazolidin-5-ones **12a**–**12c**.

Entry	Reactants	R	Isoxazolidinol	Yield ^1^	dr ^2^	Isoxazolidinone	Yield ^1^
1	**7a, 10**	Me	**11a**	83%	2:1	**12a**	98%
2	**7b, 10**	PhCH_2_	**11b**	79%	3:1	**12b**	99%
3	**7c, 10**	*n*-Hexyl	**11c**	78%	3:1	**12c**	87%

^1^ Yield of isolated product after chromatographic purification. ^2^ By ^1^H-NMR.

**Table 3:** Enantioselective synthesis of pyrazolidin-3-ones **9**.

Entry	Reactants	Catalyst	Pyrazolidinol	Yield ^1^	dr ^2^	Pyrazolidinone	Yield ^1^	er ^3^
1	**7a, 2a**	**13**	**8aa**	82%	4:1	**9aa**	87%	50:50
2	**7a, 2a**	**14**	**8aa**	99%	3:1	**9aa**	94%	90:10
3	**7b, 2a**	**13**	**8ab**	77%	>20:1	**9ab**	98%	44:56
4	**7b, 2a**	**14**	**8ab**	98%	>20:1	**9ab**	98%	45:55
5	**7b, 2b**	**13**	**8bb**	85%	9:1	**9bb**	97%	- ^4^
6	**7b, 2b**	**14**	**8bb**	85%	9:1	**9bb**	97%	- ^4^
7	**7a, 2b**	**13**	**8ba**	84%	8:1	**9ba**	98%	96:4
8	**7a, 2b**	**14**	**8ba**	0% ^5^		**9ba**	-	-
9	**7c, 2b**	**13**	**8bc**	93%	10:1	**9bc**	98%	78:22
10	**7c, 2b**	**14**	**8bc**	77%	10:1	**9bc**	98%	50:50

^1^ Yield of isolated product after chromatographic purification; ^2^ By ^1^H-NMR; ^3^ By chiral HPLC analysis (See the Supplementary Materials for details); ^4^ No satisfactory conditions for the HPLC analysis of this compound could be found; ^5^ No reaction was observed after 7 days.

**Table 4:** Active compounds ^1^ in the primary antimicrobial screening (bacterial and fungal).

Entry	*S. aureus*	*E. coli*	*K. pneumoniae*	*A. baumannii*	*P. aeruginosa*	*C. albicans*	*C. neoformans*
1	-	-	-	-	-	**12b**	-
2	**8aa** (*rac*)	-	-	-	-	-	-
3	**8aa** (90:10 er)	-	-	-	-	-	-
4	-	-	-	-	-	-	**12a**
5	-	-	-	-		**11c**	-

^1^ Compounds showing inhibition > 80% and Z-score > 2.5 in two replicate experiments, at a concentration of 32 μg/mL (water/DMSO).

## Supplementary Materials

The following are available online at: http://www.mdpi.com/1420-3049/21/12/1655/s1, NMR spectra and chiral HPLC traces for compounds **3**, **5**, **8aa**–**8bc**, **9aa**–**9bc**, **11a**–**11c** and **12a**–**12c** (31 pages).
