# Supplementary material for: Expedient Organocatalytic Syntheses of 4-Substituted Pyrazolidines and Isoxazolidines
Source: Molecules. 2016 Dec 1;21(12):1655. doi: 10.3390/molecules21121655 (PMC6274280; doi:10.3390/molecules21121655)
Supplement: Supplementary file 1 [file molecules-21-01655-s001.pdf]

Molecules **2016**, *21*, 1655; doi:10.3390/molecules21121655

Supplementary Materials: Expedient Organocatalytic Syntheses of 4-Substituted Pyrazolidines and Isoxazolidines

Tarek Yousfi, Alysha Elliott, Messiad Hanane, Rachid Merdes and Albert Moyano

NMR spectra and chiral HPLC traces for compounds 3, 5, 8aa–8bc, 9aa–9bc, 11a–11c and 12a–12c.

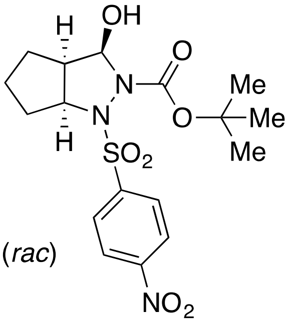

3 (*rac*)

Figure S1. *tert*-Butyl (3*RS*,3a*RS*,6a*SR*)-3-Hydroxy-1-((4-nitrophenyl)sulfonyl) hexahydrocyclopenta [*c*] pyrazole-2(1*H*)-carboxylate, **3**.

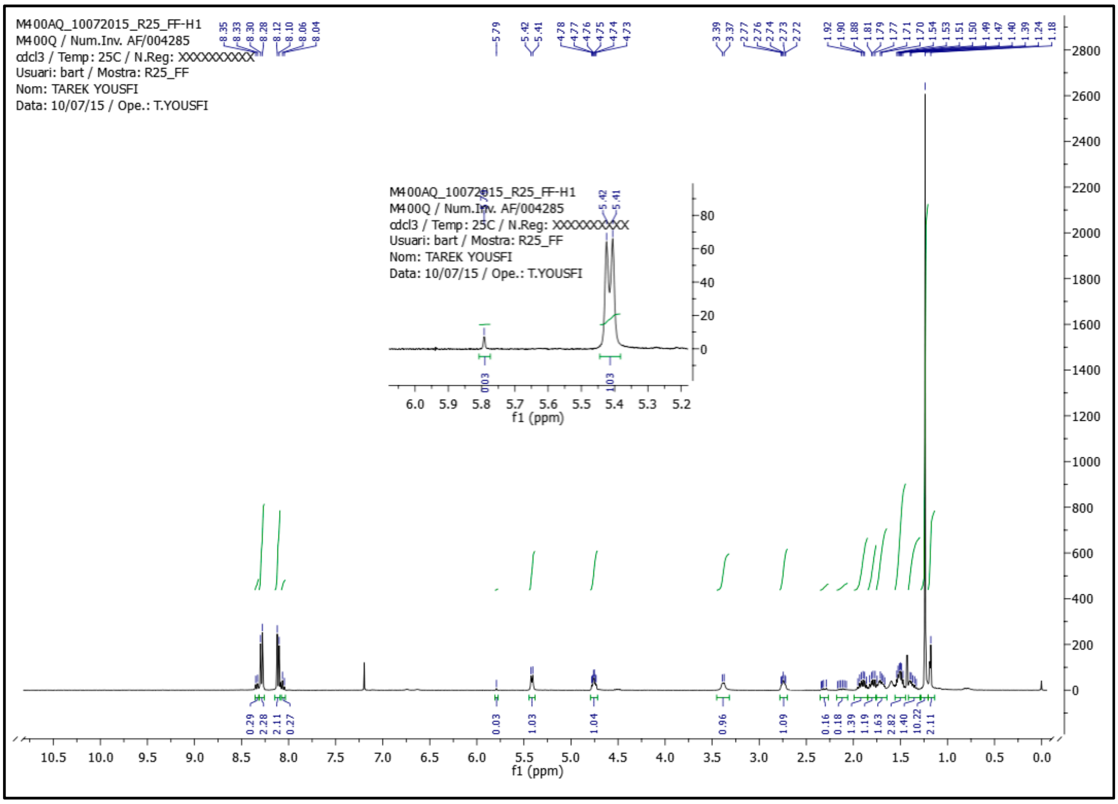

Figure S2. ^{1}H-NMR (400 MHz) of *tert*-Butyl (3*RS*,3a*RS*,6a*SR*)-3-Hydroxy-1-((4-nitrophenyl)sulfonyl) hexahydrocyclopenta [*c*] pyrazole-2(1*H*)-carboxylate, **3**.

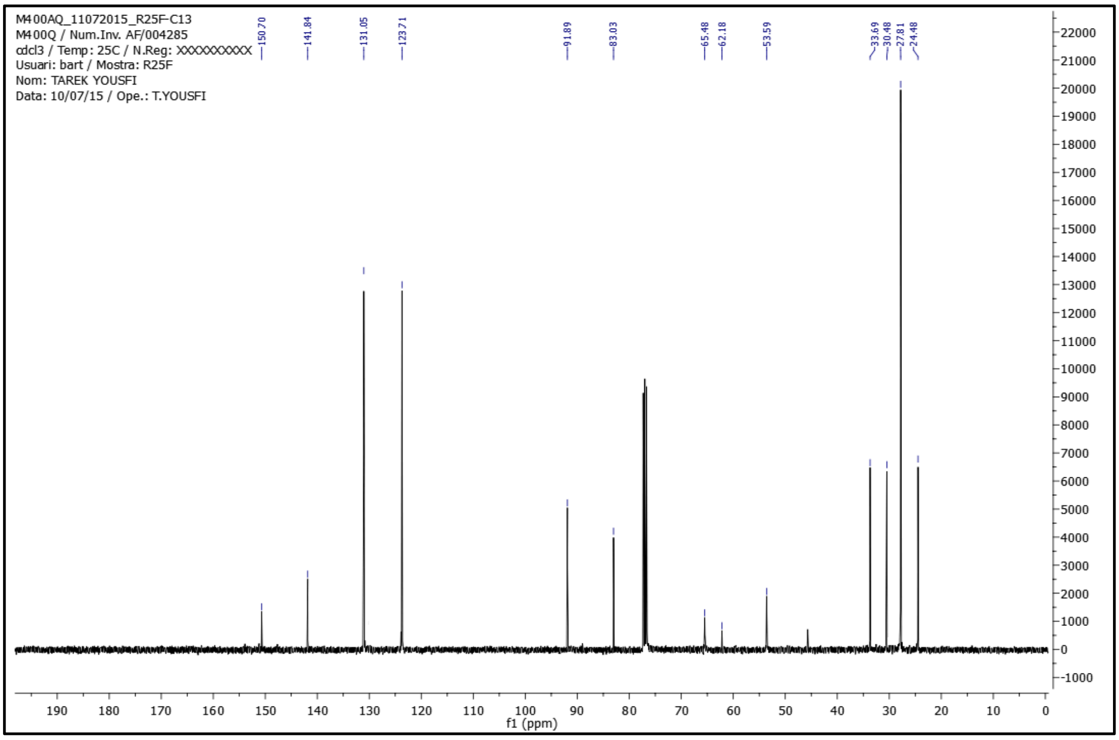

Figure S3. ^{13}C-NMR (100.6 MHz) of *tert*-Butyl (3*RS*,3a*RS*,6a*SR*)-3-Hydroxy-1-((4-nitrophenyl)sulfonyl) hexahydrocyclopenta [*c*] pyrazole-2(1*H*)-carboxylate, **3**.

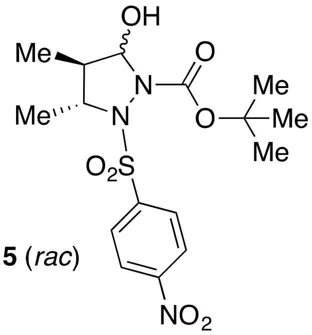

5 (*rac*)

Figure S4. *tert*-Butyl 3,4-*trans*-5-Hydroxy-3,4-dimethyl-2-((4-nitrophenyl)sulfonyl)-pyrazolidine-1-carboxylate, **5**.

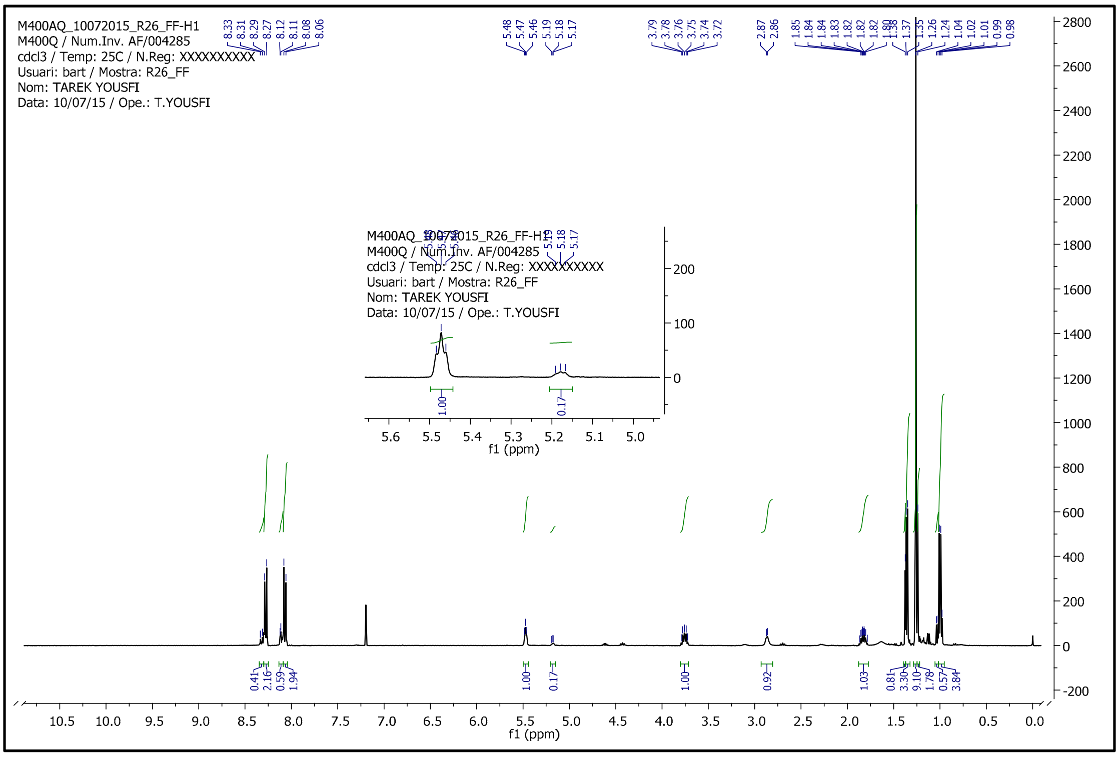

Figure S5. ^{1}H-NMR (400 MHz) of *tert*-Butyl 3,4-*trans*-5-Hydroxy-3,4-dimethyl-2-((4-nitrophenyl)sulfonyl)-pyrazolidine-1-carboxylate, **5**.

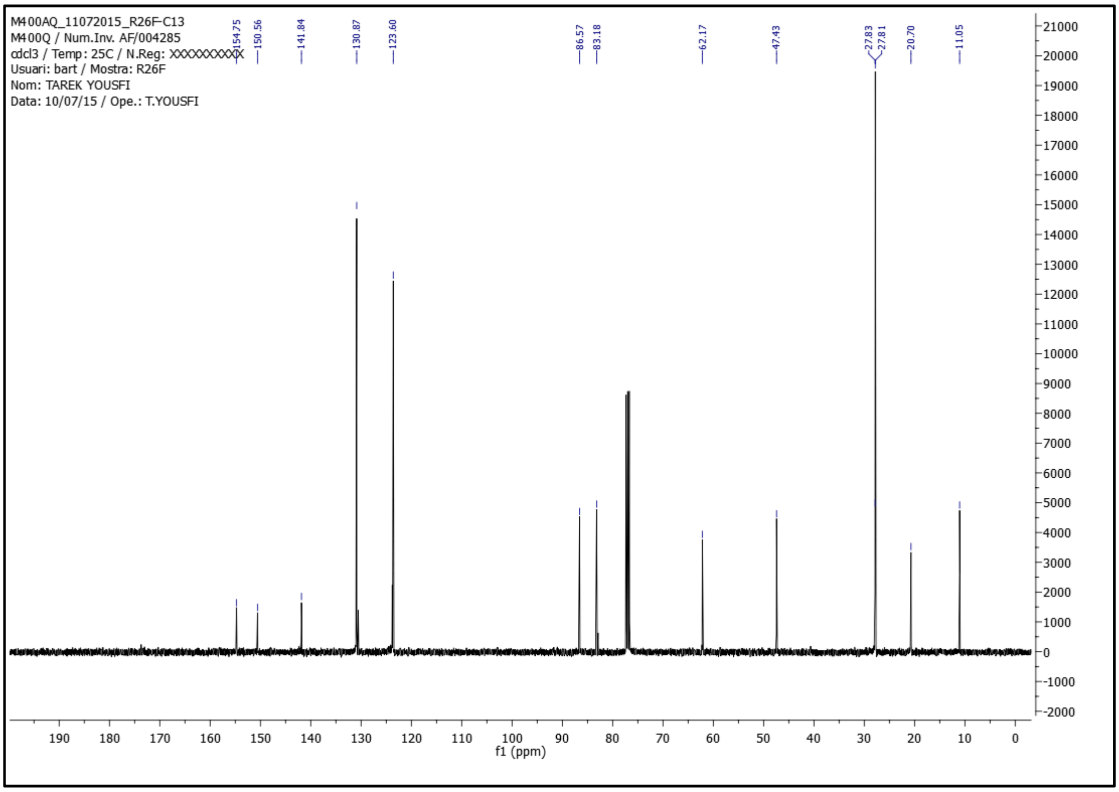

Figure S6. ^{13}C-NMR (100.6 MHz) of *tert*-Butyl 3,4-*trans*-5-Hydroxy-3,4-dimethyl-2-((4-nitrophenyl)sulfonyl)-pyrazolidine-1-carboxylate, **5**.

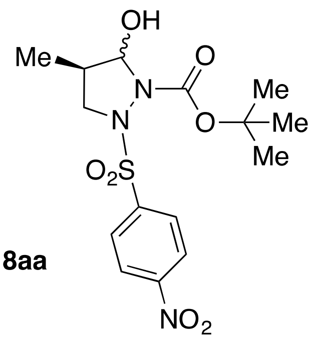

8aa

Figure S7. *tert*-Butyl 5-Hydroxy-4-methyl-2-((4-nitrophenyl)sulfonyl)-pyrazolidine-1-carboxylate, **8aa**.

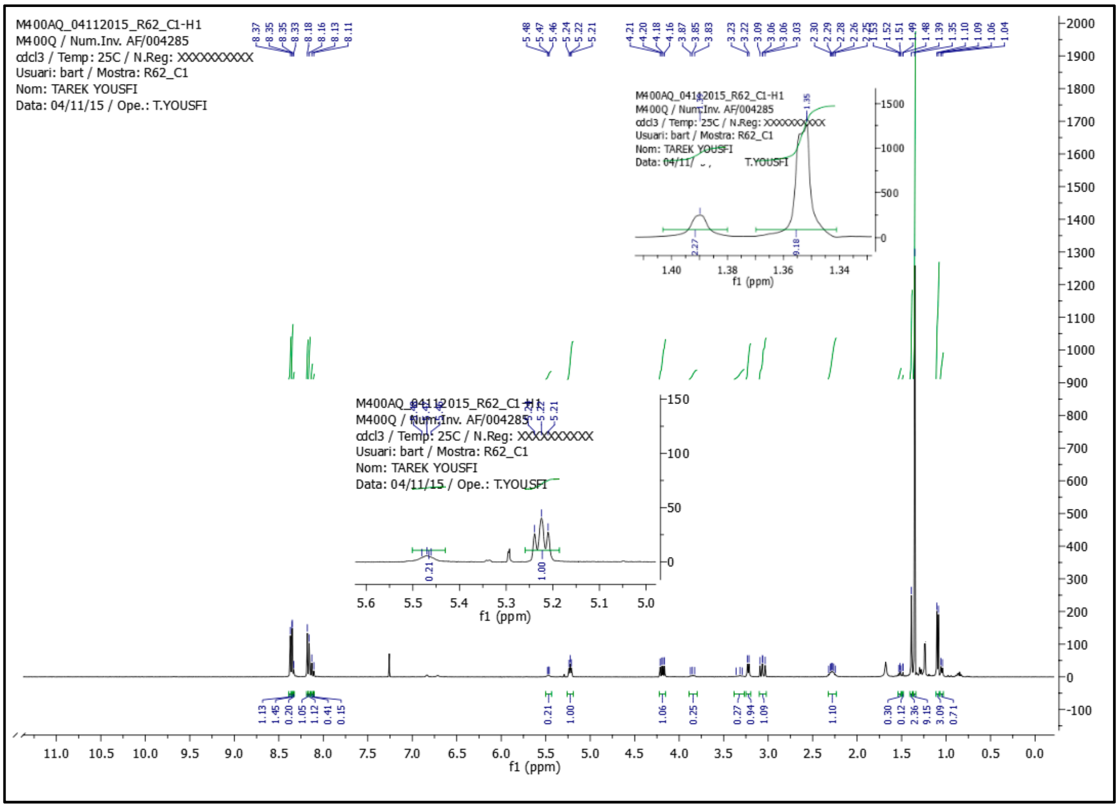

Figure S8. ^{1}H-NMR (400 MHz) of *tert*-Butyl 5-Hydroxy-4-methyl-2-((4-nitrophenyl)sulfonyl)-pyrazolidine-1-carboxylate, **8aa**.

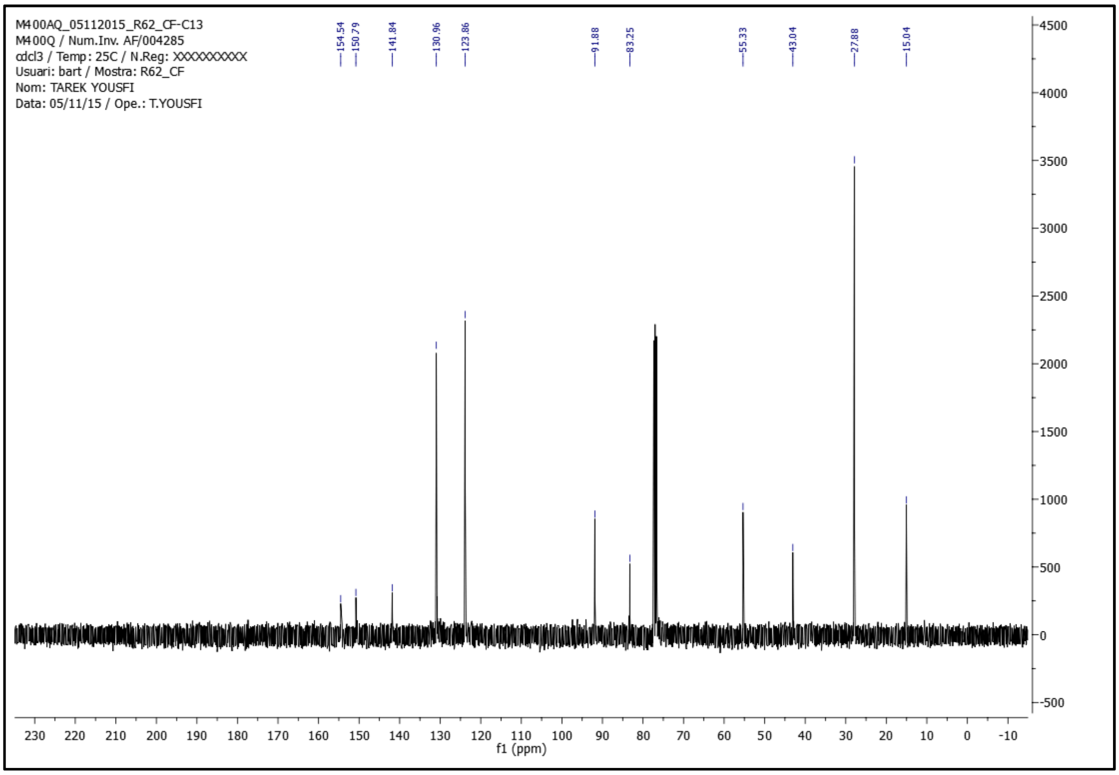

Figure S9. ^{13}C-NMR (100.6 MHz) of *tert*-Butyl 5-Hydroxy-4-methyl-2-((4-nitrophenyl)sulfonyl)-pyrazolidine-1-carboxylate, **8aa**.

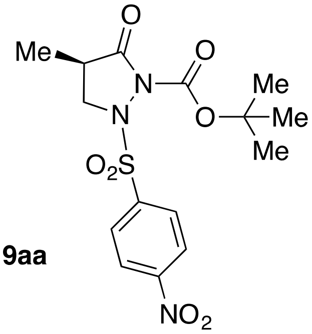

9aa

Figure S10. (−)-*tert*-Butyl 4-Methyl-2-((4-nitrophenyl)sulfonyl)-pyrazolidin-5-one-1-carboxylate, **9aa**.

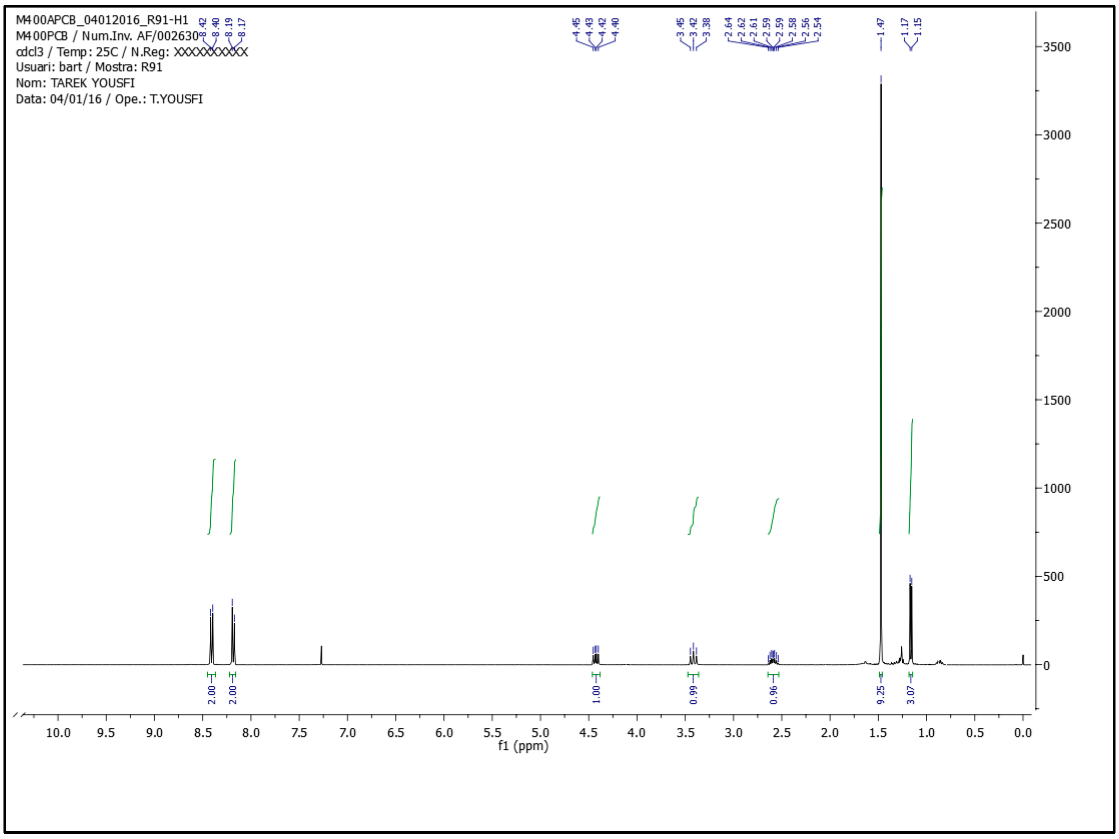

Figure S11. ^{1}H-NMR (400 MHz) of (−)-*tert*-Butyl 4-Methyl-2-((4-nitrophenyl)sulfonyl)-pyrazolidin-5-one-1-carboxylate, **9aa**.

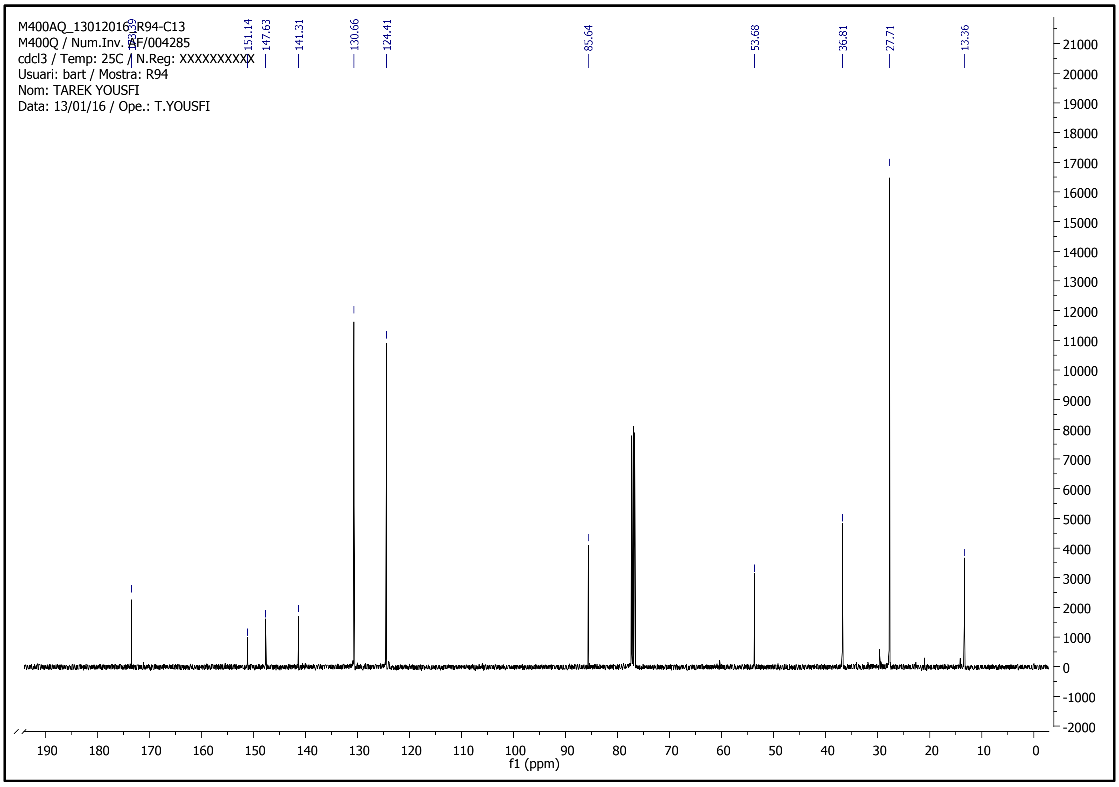

Figure S12. ^{13}C-NMR (100.6 MHz) of (−)-*tert*-Butyl 4-Methyl-2-((4-nitrophenyl)sulfonyl)-pyrazolidin-5-one-1-carboxylate, **9aa**.

HPLC (Chiralpak® IC column, 90:10 hexane/isopropyl alcohol, 1 μL/min, λ = 254 nm, 25 °C).

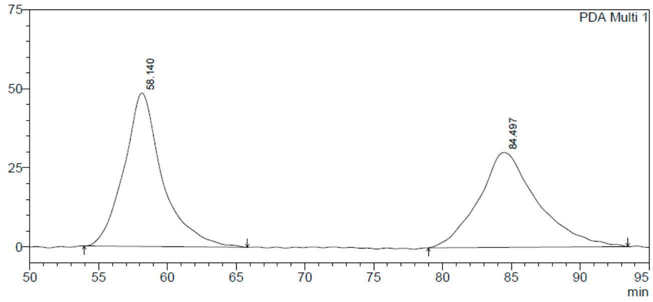

PDA Ch1 254nm 4nm

Peak#	Ret. Time	Area	Height	Area %	Height %
1	58.140	9422214	48509	51.307	61.789
2	84.497	8942203	29999	48.693	38.211
Total		18364416	78508	100.000	100.000

Figure S13. *rac*-**9aa**.

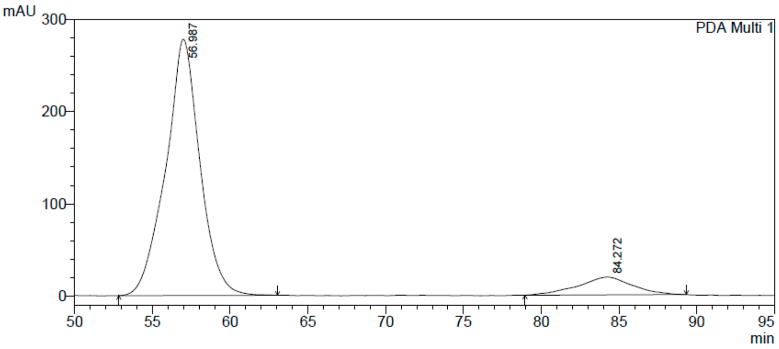

Peak#	Ret. Time	Area	Height	Area %	Height %
1	56.987	42488086	277822	90.079	93.548
2	84.272	4679622	19162	9.921	6.452
Total		47167708	296984	100.000	100.000

Figure S14. (−)-**9aa**.

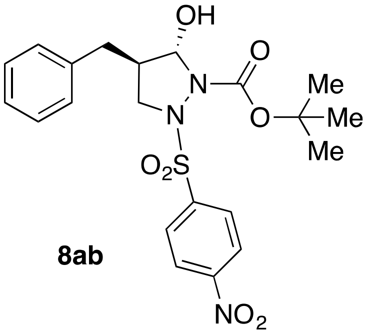

8ab

Figure S15. *tert*-Butyl 4-Benzyl-5-hydroxy-2-((4-nitrophenyl)sulfonyl)-pyrazolidine-1-carboxylate, **8ab**.

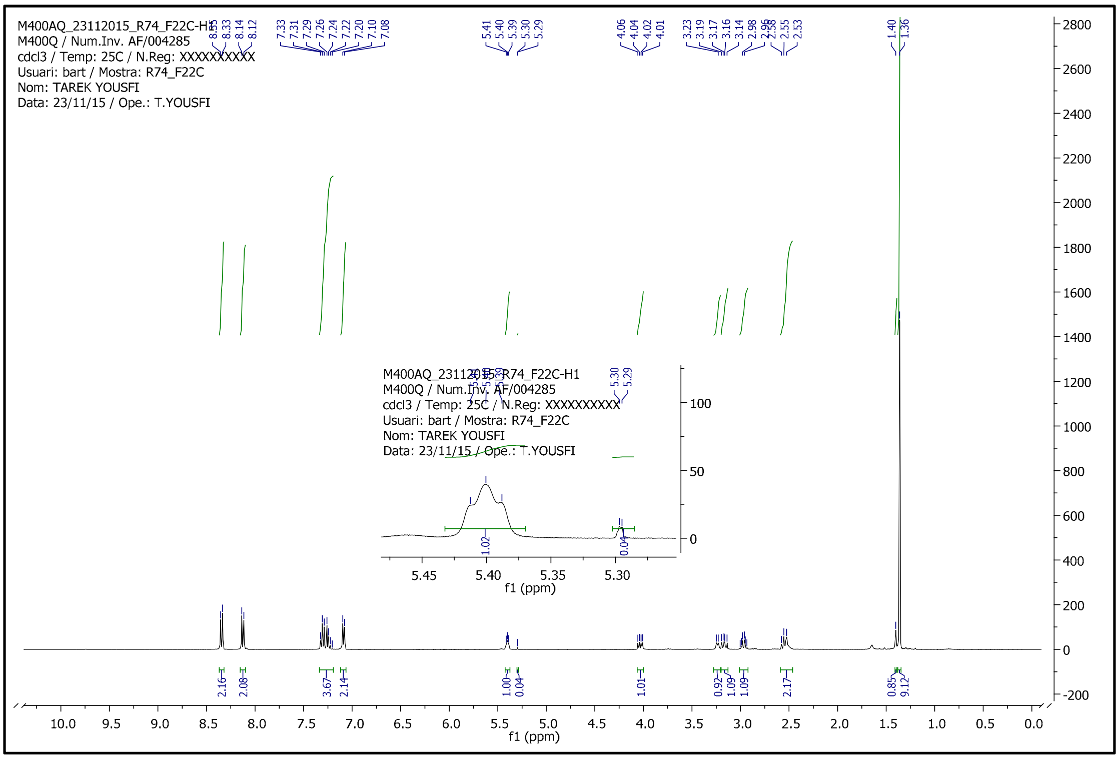

Figure S16. ^{1}H-NMR (400 MHz) of *tert*-Butyl 4-Benzyl-5-hydroxy-2-((4-nitrophenyl)sulfonyl)-pyrazolidine-1-carboxylate, **8ab**.

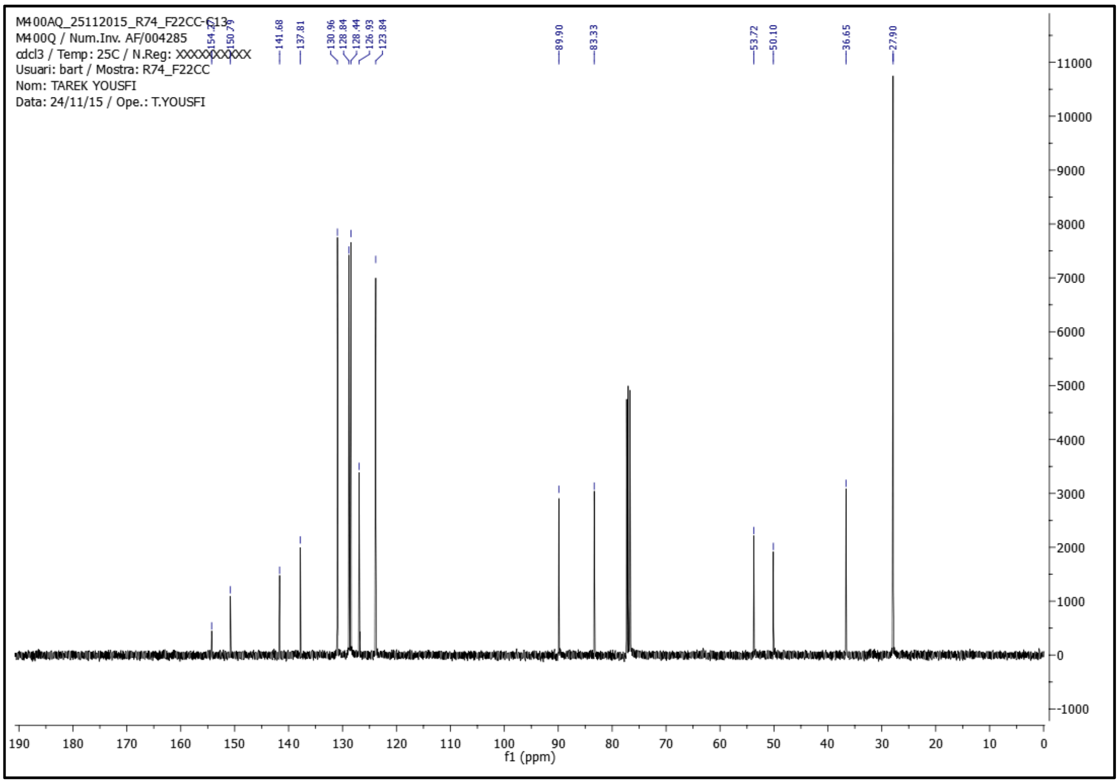

Figure S17. ^{13}C-NMR (100.6 MHz) of *tert*-Butyl 4-Benzyl-5-hydroxy-2-((4-nitrophenyl)sulfonyl)-pyrazolidine-1-carboxylate, **8ab**.

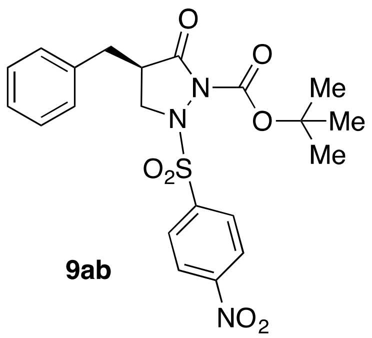

9ab

Figure S18. (−)-*tert*-Butyl 4-Benzyl-2-((4-nitrophenyl)sulfonyl)-pyrazolidin-5-one-1-carboxylate, **9ab**.

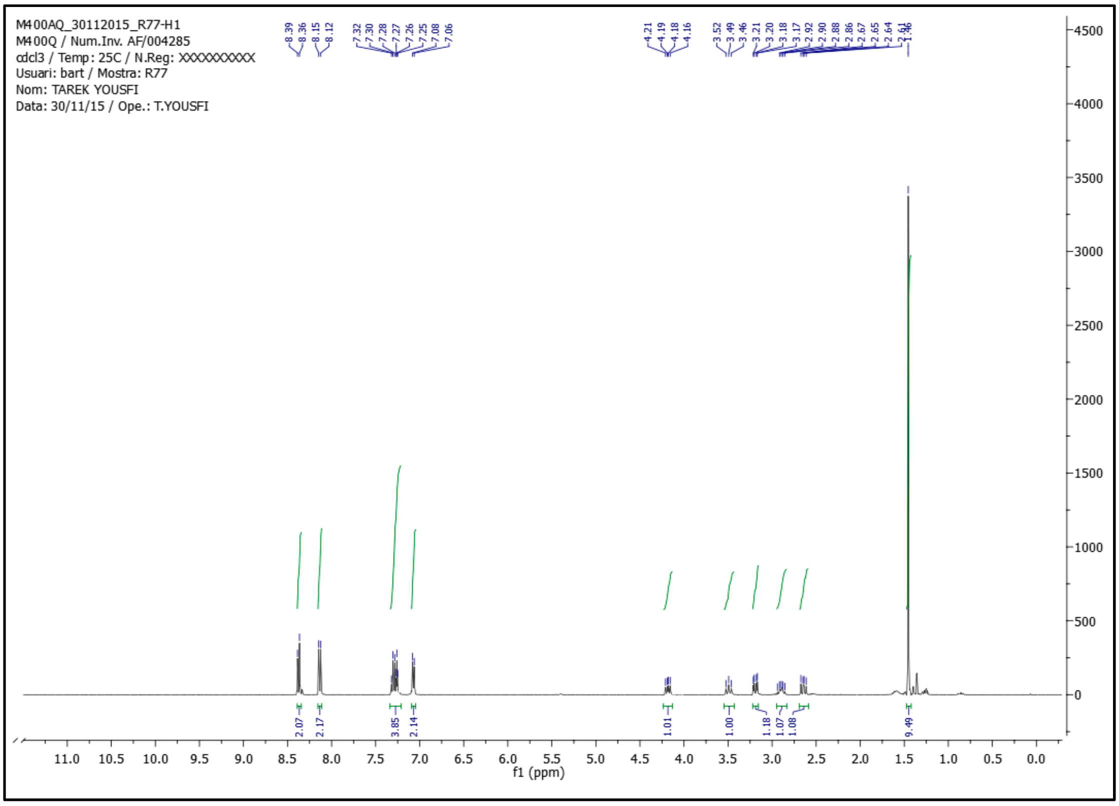

Figure S19. ^{1}H-NMR (400 MHz) of (−)-*tert*-Butyl 4-Benzyl-2-((4-nitrophenyl)sulfonyl)-pyrazolidin-5-one-1-carboxylate, **9ab**.

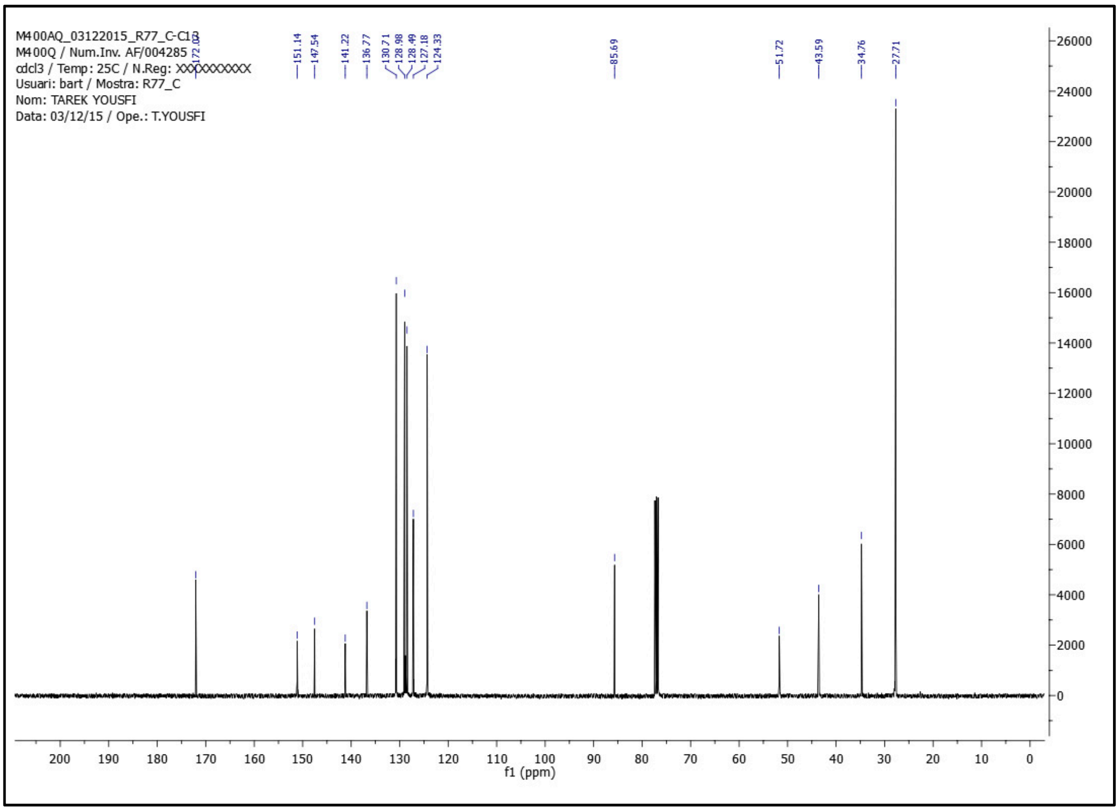

Figure S20. ^{13}C-NMR (100.6 MHz) of (−)-*tert*-Butyl 4-Benzyl-2-((4-nitrophenyl)sulfonyl)-pyrazolidin-5-one-1-carboxylate, **9ab**.

HPLC (Chiralpak® IC column, 90:10 hexane/isopropyl alcohol, 1 μL/min, λ = 254 nm, 25 °C).

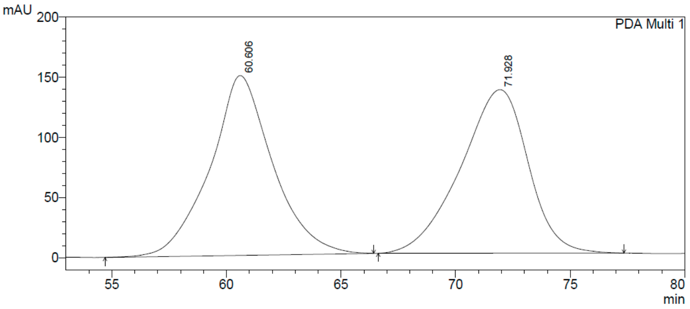

Peak#	Ret. Time	Area	Height	Area %	Height %
1	60.606	28447344	149356	50.513	52.370
2	71.928	27870083	135839	49.487	47.630
Total		56317428	285195	100.000	100.000

Figure S21. *rac*-**9ab**.

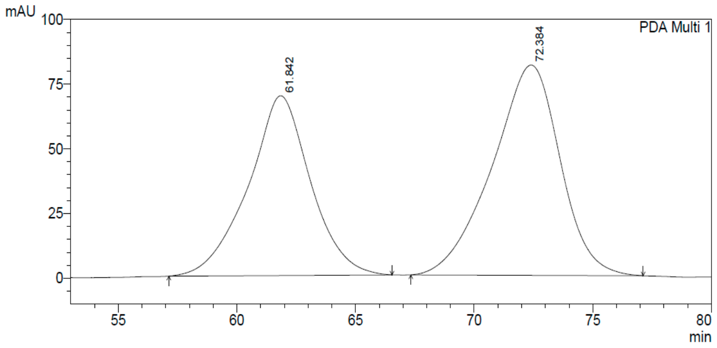

Peak#	Ret. Time	Area	Height	Area %	Height %
1	61.842	12677296	69502	43.890	46.085
2	72.384	16207129	81310	56.110	53.915
Total		28884425	150813	100.000	100.000

Figure S22. (−)-**9ab**.

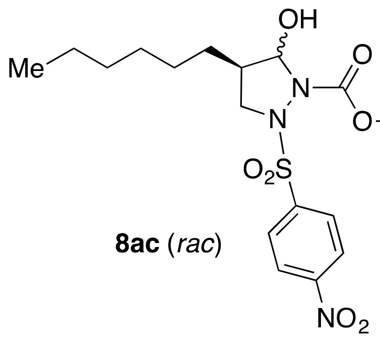

8ac (*rac*)

Figure S23. *tert*-Butyl 4-Hexyl-5-hydroxy-2-((4-nitrophenyl)sulfonyl)-pyrazolidine-1-carboxylate, **8ac**.

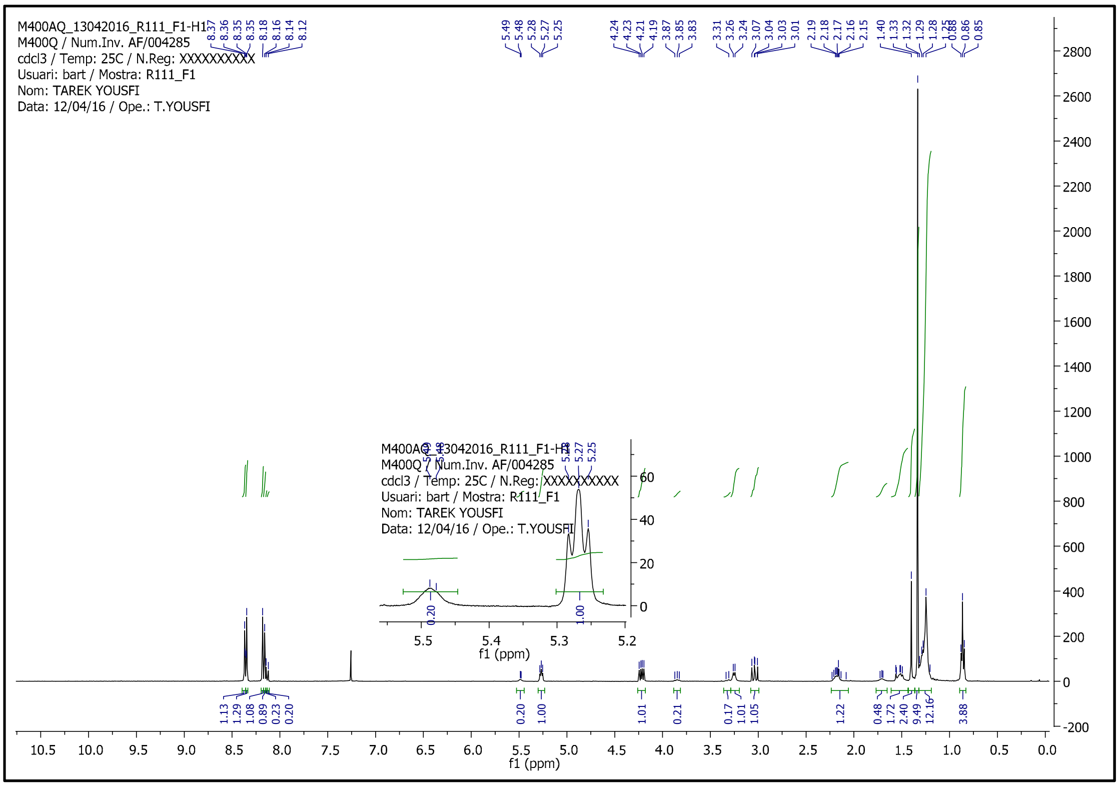

Figure S24. ^{1}H-NMR (400 MHz) of *tert*-Butyl 4-Hexyl-5-hydroxy-2-((4-nitrophenyl)sulfonyl)-pyrazolidine-1-carboxylate, **8ac**.

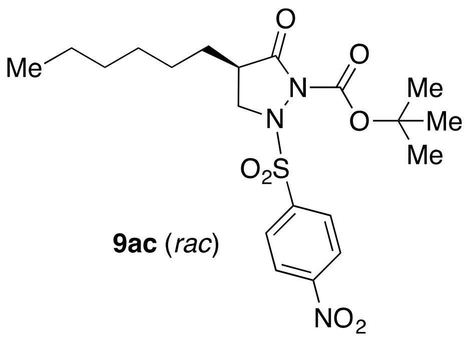

Figure S25. *tert*-Butyl 4-Hexyl-2-((4-nitrophenyl)sulfonyl)-pyrazolidin-5-one-1-carboxylate, **9ac**.

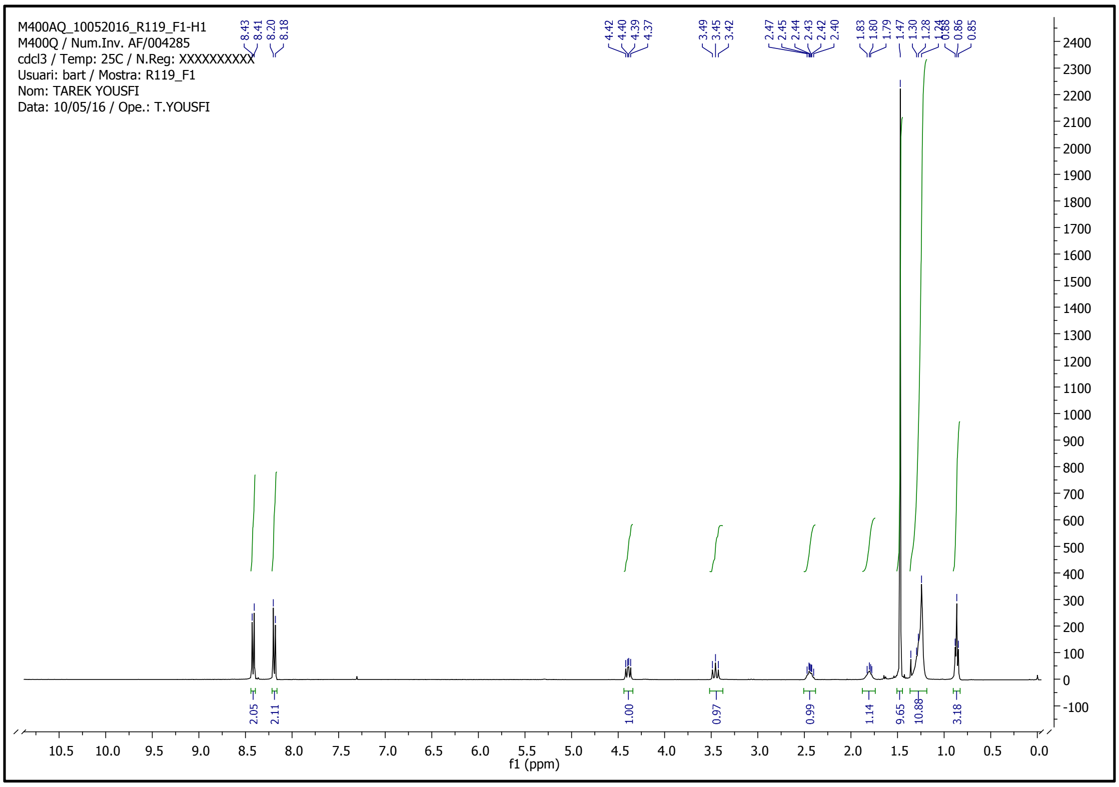

Figure S26. ^{1}H-NMR (400 MHz) of *tert*-Butyl 4-Hexyl-2-((4-nitrophenyl)sulfonyl)-pyrazolidin-5-one-1-carboxylate, **9ac**.

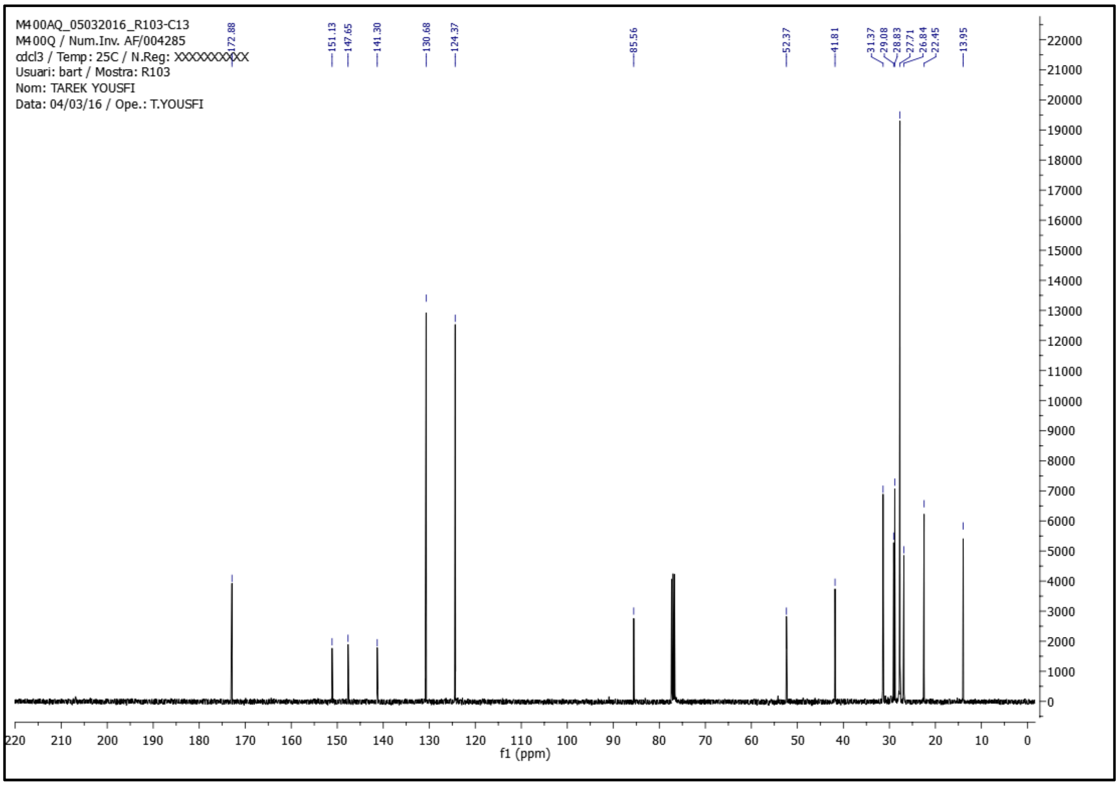

Figure S27. ^{13}C-NMR (100.6 MHz) of *tert*-Butyl 4-Hexyl-2-((4-nitrophenyl)sulfonyl)-pyrazolidin-5-one-1-carboxylate, **9ac**.

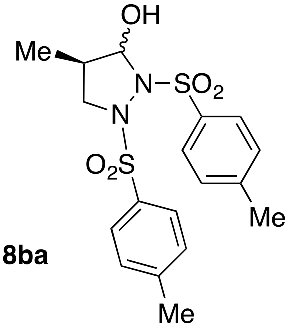

8ba

Figure S28. 4-Methyl-1,2-bis-(4-toluenesulfonyl)-pyrazolidin-3-ol, **8ba**.

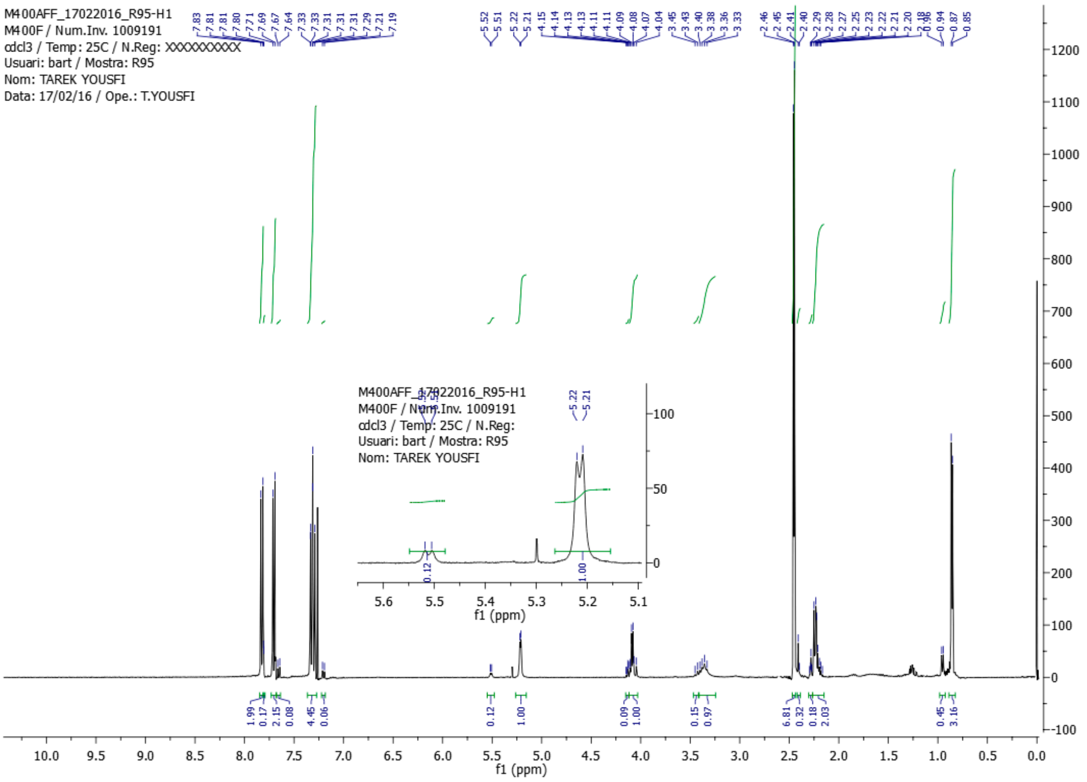

Figure S29. ^{1}H-NMR (400 MHz) of 4-Methyl-1,2-bis-(4-toluenesulfonyl)-pyrazolidin-3-ol, **8ba**.

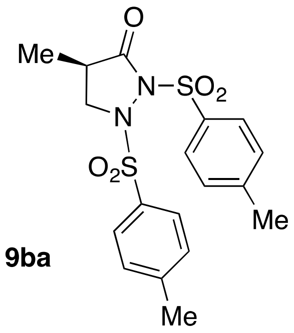

Figure S30. (−)-4-Methyl-1,2-bis-(4-toluenesulfonyl)-pyrazolidin-3-one, **9ba**.

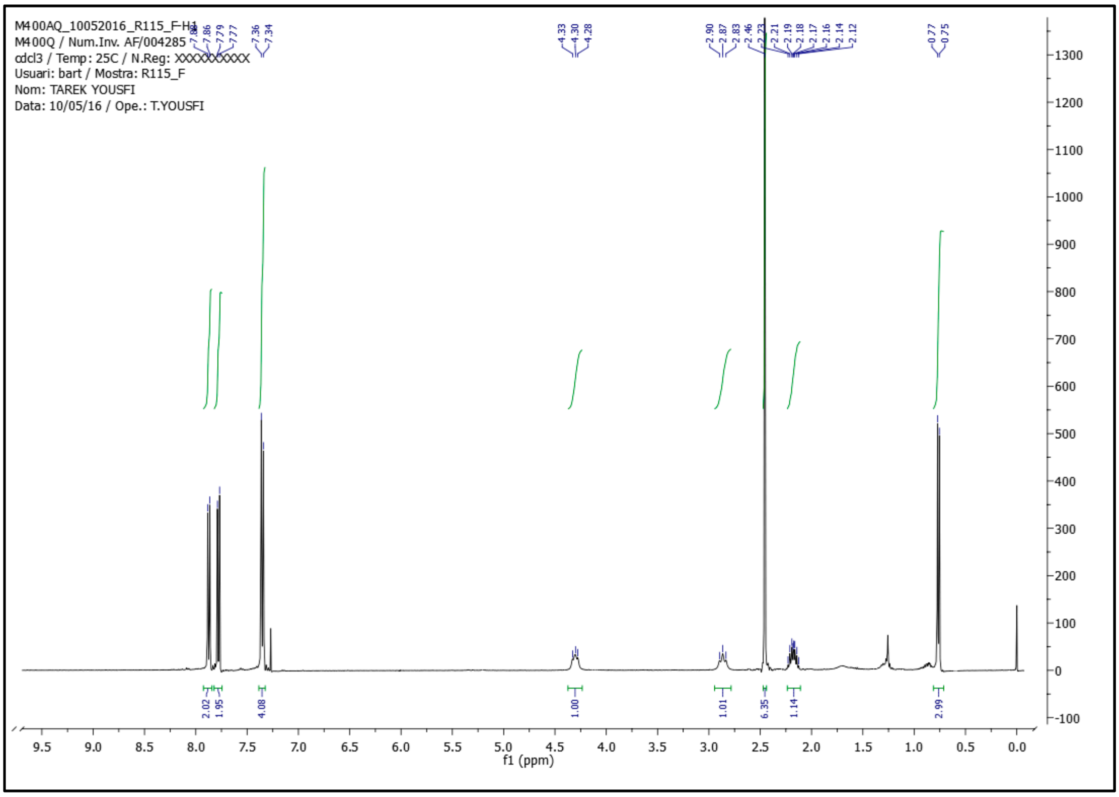

Figure S31. ^{1}H-NMR (400 MHz) of (−)-4-Methyl-1,2-bis-(4-toluenesulfonyl)-pyrazolidin-3-one, **9ba**.

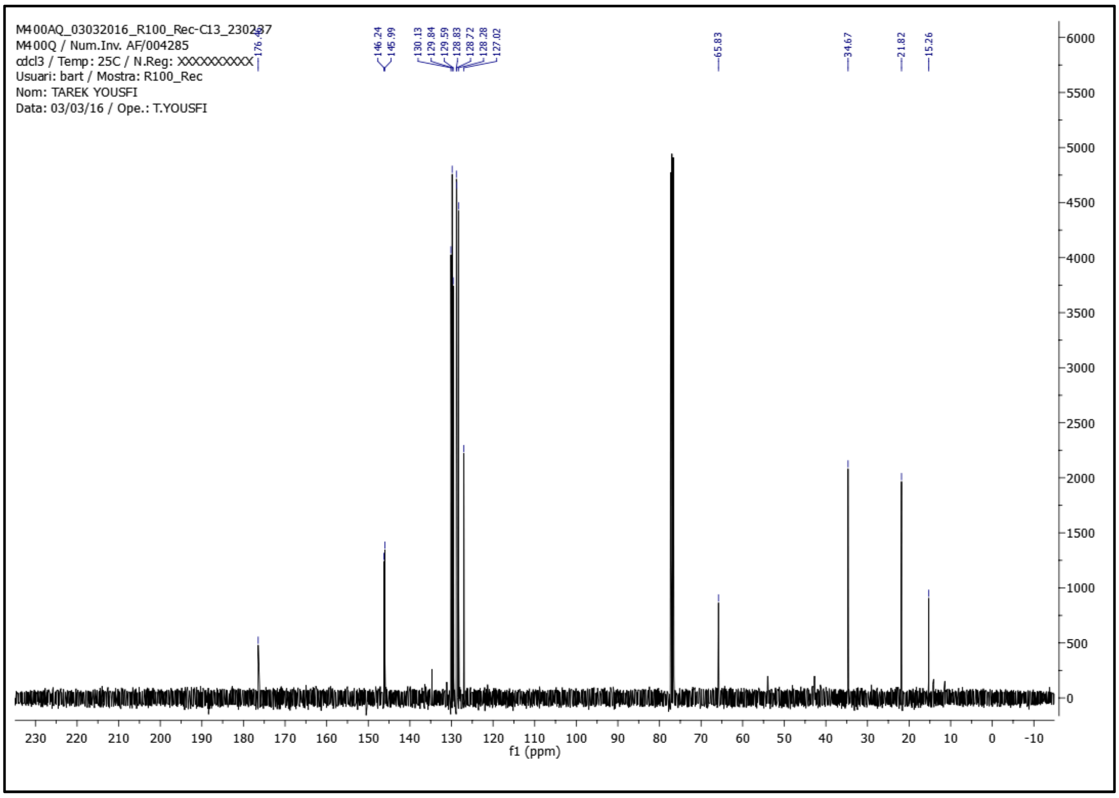

Figure S32. ^{13}C-NMR (100.6 MHz) of (−)-4-Methyl-1,2-bis-(4-toluenesulfonyl)-pyrazolidin-3-one, **9ba**.

HPLC (Chiralpak® IA column, 90:10 hexane/isopropyl alcohol, 1 µL/min, λ = 254 nm, 25 °C).

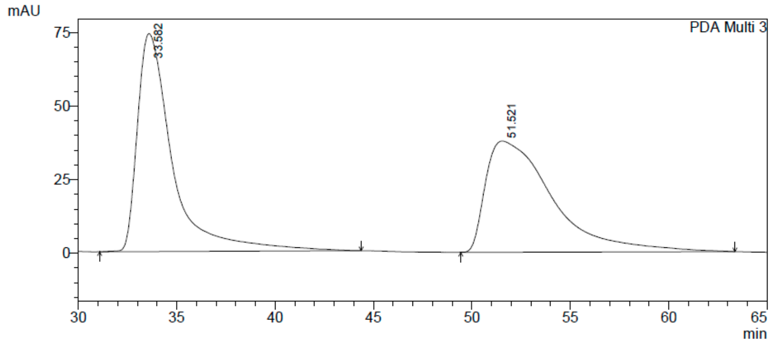

PDA Ch3 254nm 4nm

Peak#	Ret. Time	Area	Height	Area %	Height %
1	33.582	9231871	74031	50.742	66.222
2	51.521	8961982	37761	49.258	33.778
Total		18193854	111792	100.000	100.000

Figure S33. *rac*-**9ba**.

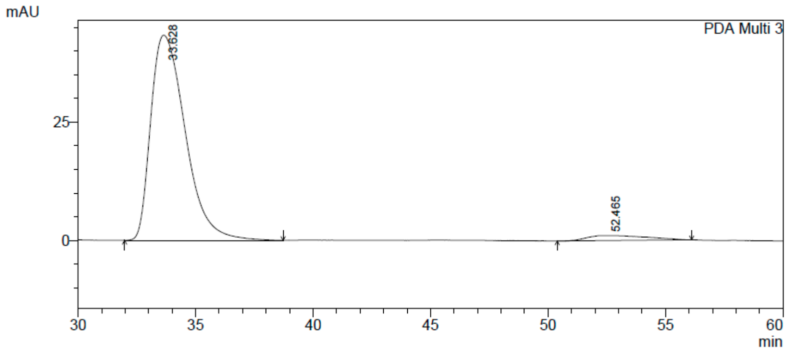

PDA Ch3 254nm 4nm

Peak#	Ret. Time	Area	Height	Area %	Height %
1	33.628	4430577	43280	95.943	97.560
2	52.465	187341	1082	4.057	2.440
Total		4617918	44362	100.000	100.000

Figure S34. (−)-**9ba**.

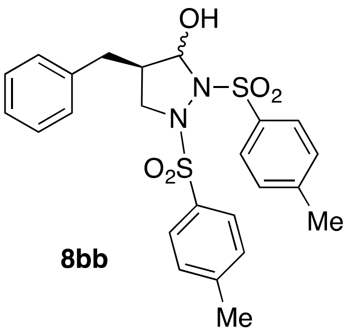

8bb

Figure S35. 4-Benzyl-1,2-bis-(4-toluenesulfonyl)-pyrazolidin-3-ol, **8bb**.

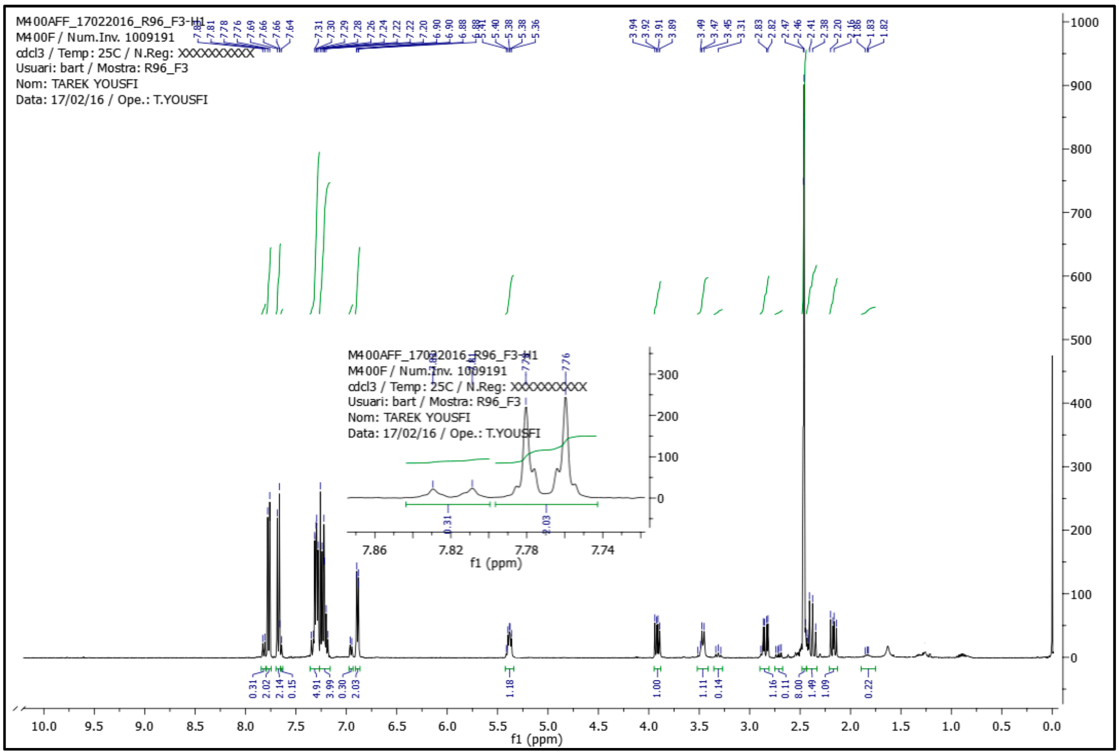

Figure S36. ^{1}H-NMR (400 MHz) of 4-Benzyl-1,2-bis-(4-toluenesulfonyl)-pyrazolidin-3-ol, **8bb**.

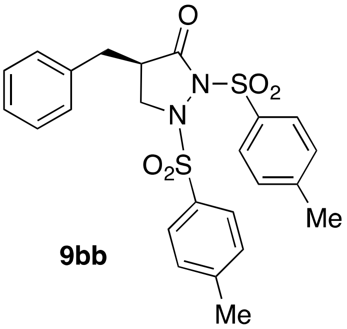

9bb

Figure S37. 4-Benzyl-1,2-bis-(4-toluenesulfonyl)-pyrazolidin-3-one, **9bb**.

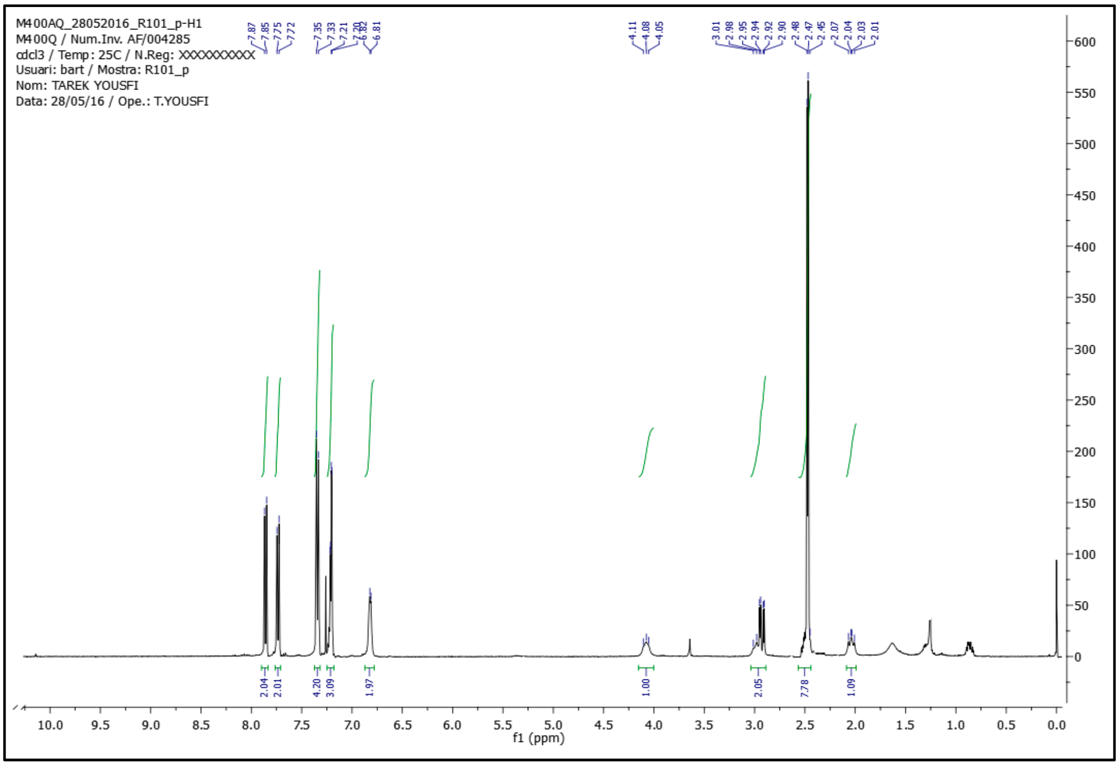

Figure S38. ^{1}H-NMR (400 MHz) of 4-Benzyl-1,2-bis-(4-toluenesulfonyl)-pyrazolidin-3-one, **9bb**.

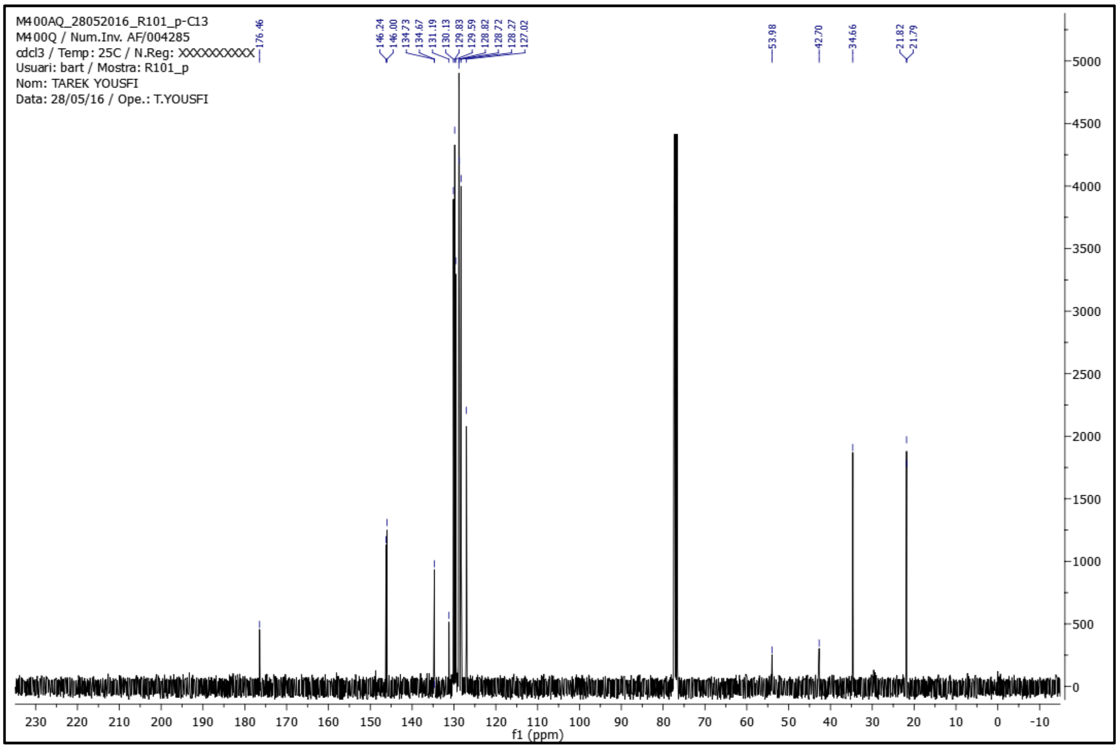

Figure S39. ^{13}C-NMR (100.6 MHz) of 4-Benzyl-1,2-bis-(4-toluenesulfonyl)-pyrazolidin-3-one, **9bb**.

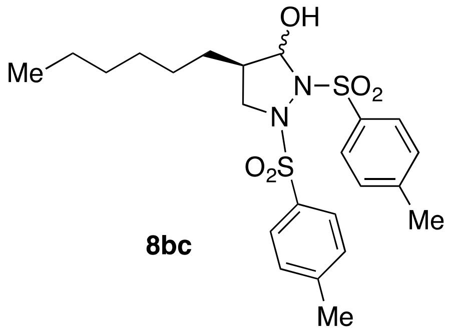

Figure S40. 4-Hexyl-1,2-bis-(4-toluenesulfonyl)-pyrazolidin-3-ol, **8bc**.

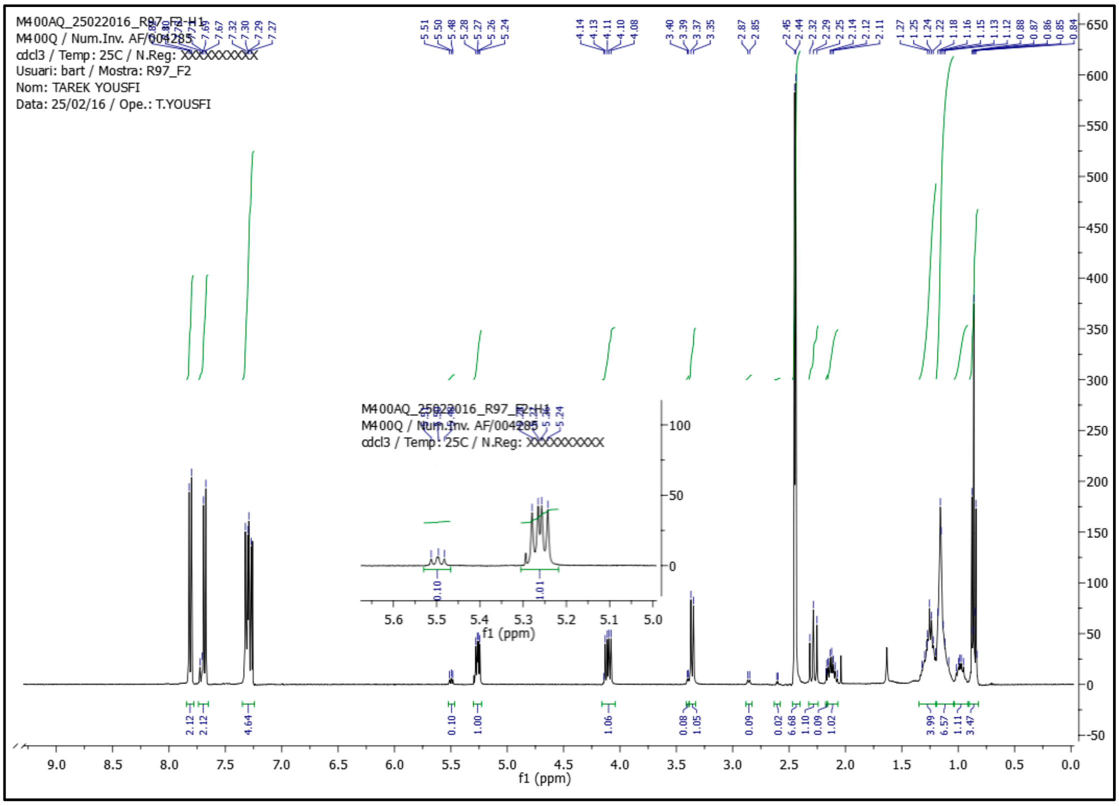

Figure S41. ^{1}H-NMR (400 MHz) of 4-Hexyl-1,2-bis-(4-toluenesulfonyl)-pyrazolidin-3-ol, **8bc**.

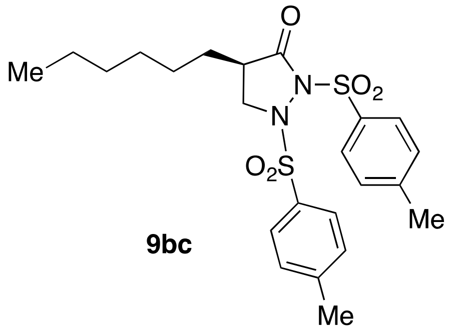

Figure S42. (−)-4-Hexyl-1,2-bis-(4-toluenesulfonyl)-pyrazolidin-3-one, **9bc**.

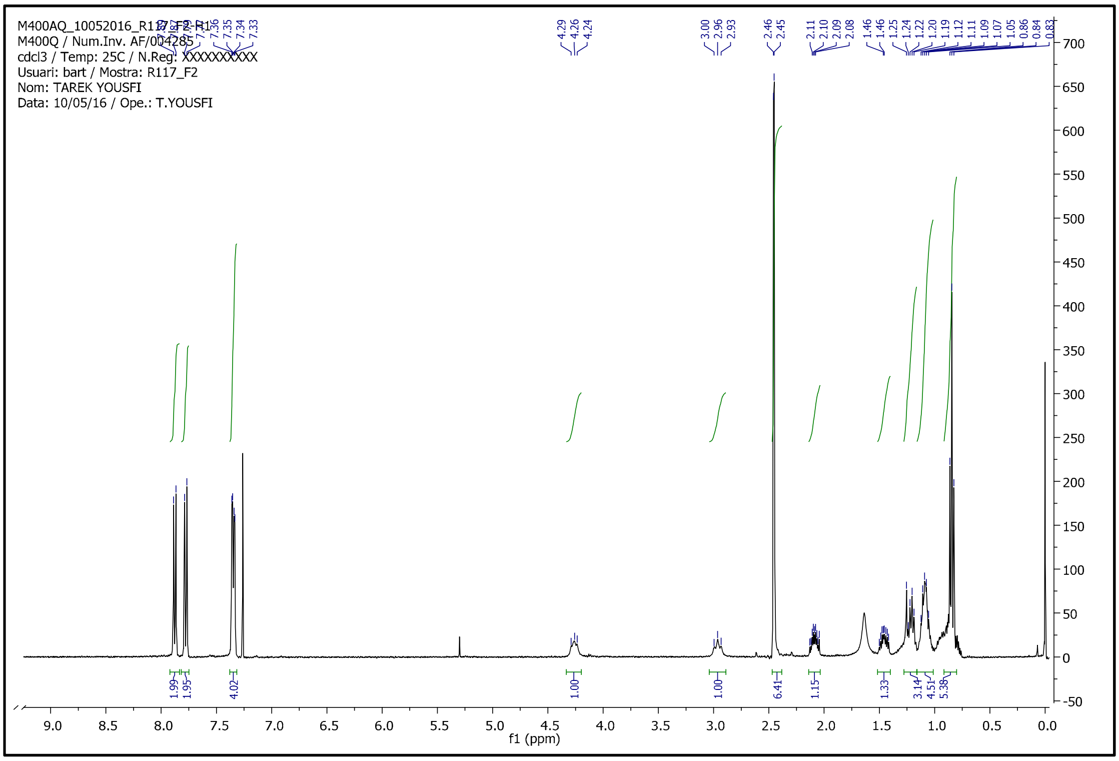

Figure S43. ^{1}H-NMR (400 MHz) of (−)-4-Hexyl-1,2-bis-(4-toluenesulfonyl)-pyrazolidin-3-one, **9bc**.

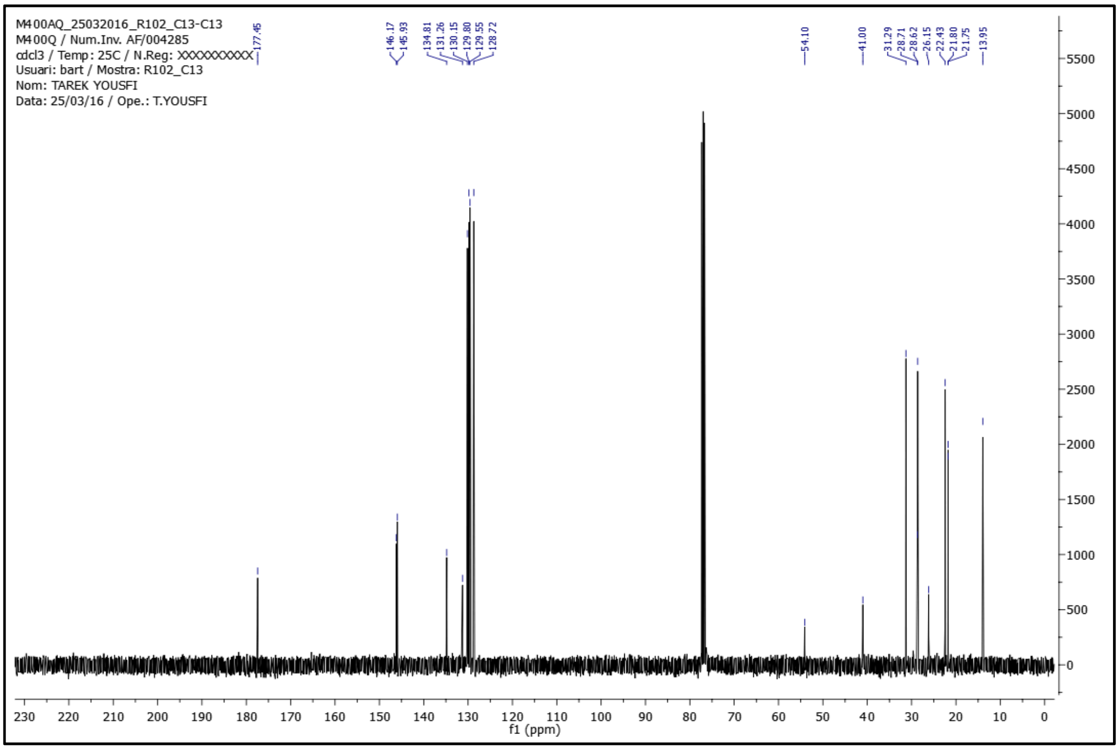

Figure S44. ^{13}C-NMR (100.6 MHz) of (−)-4-Hexyl-1,2-bis-(4-toluenesulfonyl)-pyrazolidin-3-one, **9bc**.

HPLC (Chiralpak® IB column, 93:7 hexane/isopropyl alcohol, 1 μL/min, λ = 220 nm, 25 °C).

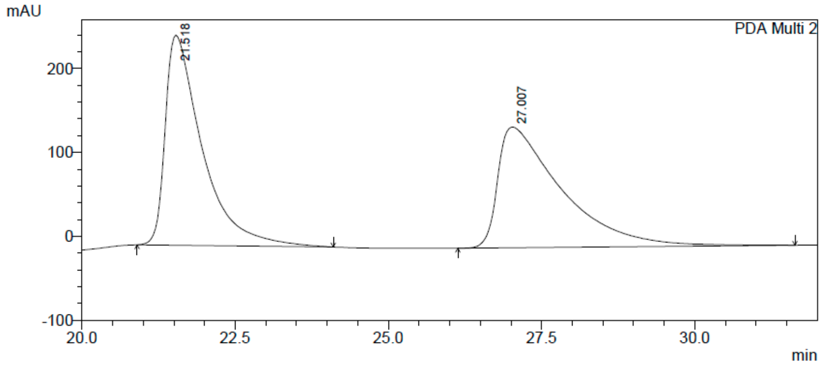

PDA Ch2 220nm 4nm

Peak#	Ret. Time	Area	Height	Area %	Height %
1	21.518	10499107	250442	50.153	63.528
2	27.007	10435081	143784	49.847	36.472
Total		20934189	394226	100.000	100.000

Figure S45. *rac*-**9bc**.

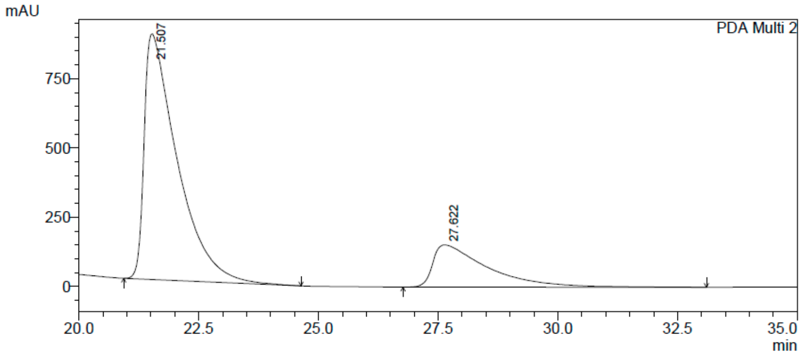

PDA Ch2 220nm 4nm

Peak#	Ret. Time	Area	Height	Area %	Height %
1	21.507	42470297	886214	77.915	85.333
2	27.622	12038479	152324	22.085	14.667
Total		54508775	1038538	100.000	100.000

Figure S46. (−)-**9bc**.

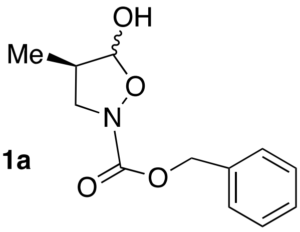

11a

Figure S47. Benzyl 5-Hydroxy-4-methyl-isoxazolidine-2-carboxylate, **11a**.

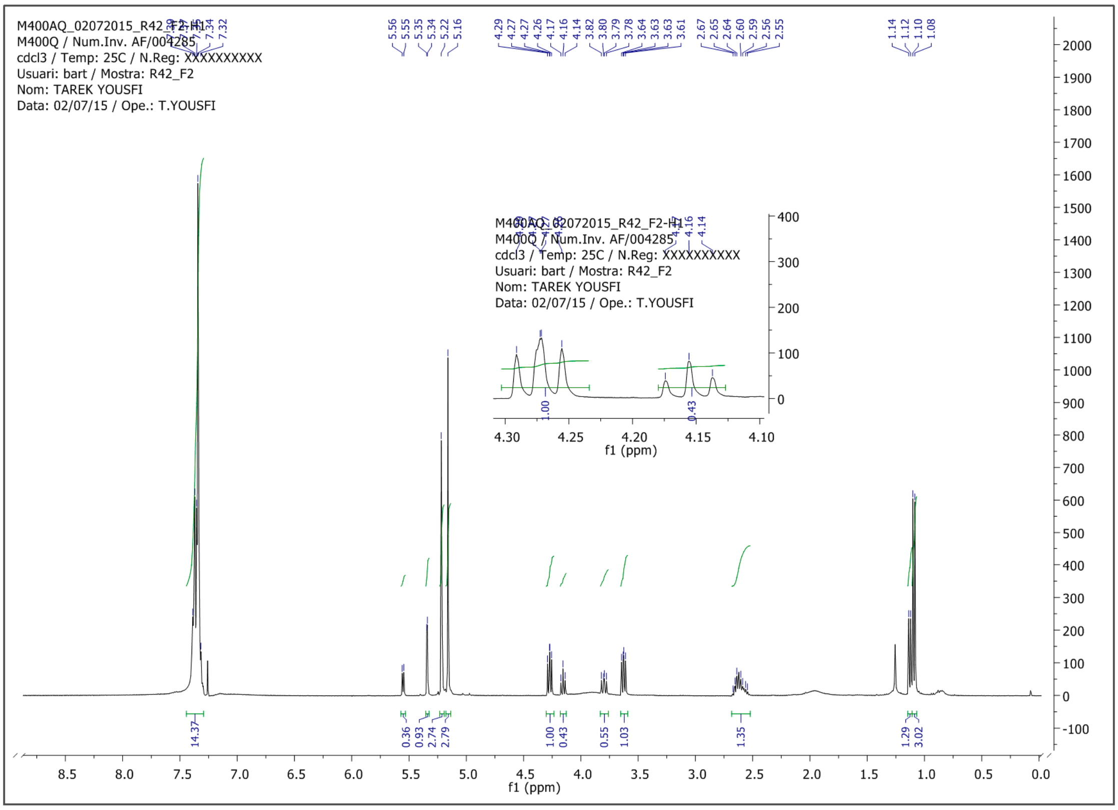

Figure S48. ^{1}H-NMR (400 MHz) of Benzyl 5-Hydroxy-4-methyl-isoxazolidine-2-carboxylate, **11a**.

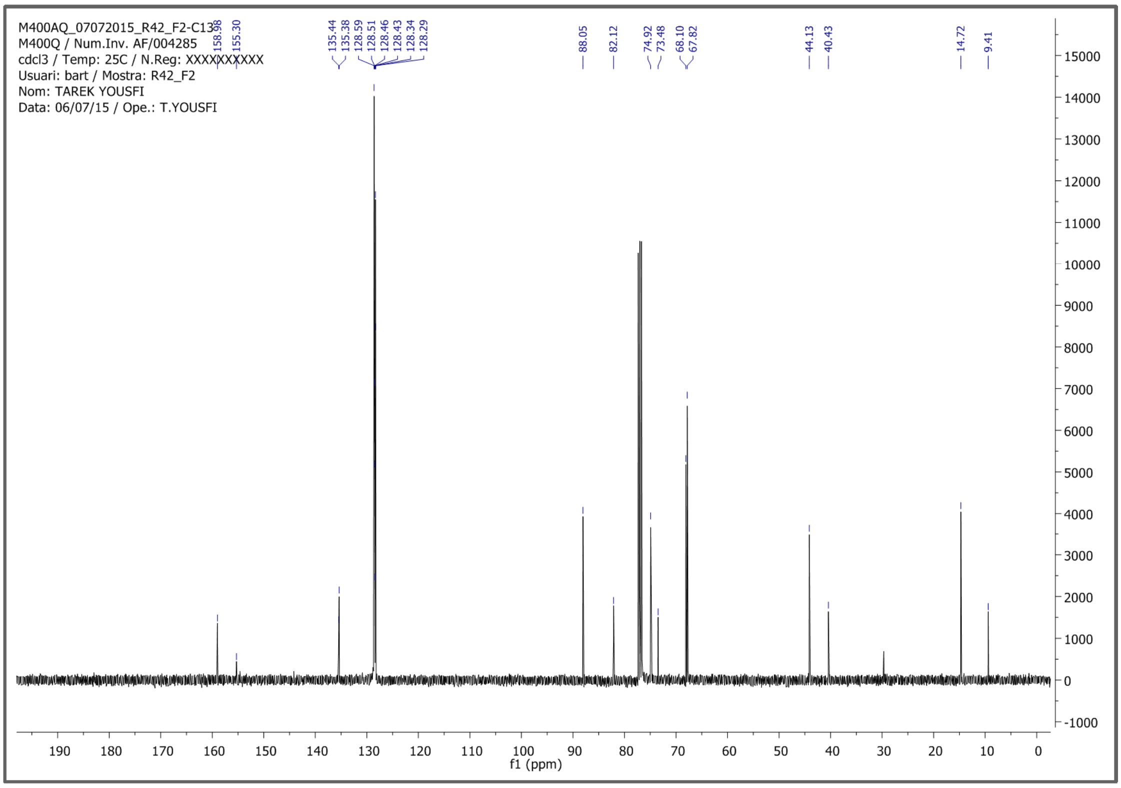

Figure S49. ^{13}C-NMR (100.6 MHz) of Benzyl 5-Hydroxy-4-methyl-isoxazolidine-2-carboxylate, **11a**.

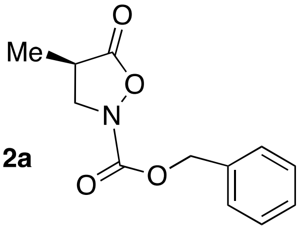

Figure S50. Benzyl 4-Methyl-5-oxo-isoxazolidine-2-carboxylate, **12a**.

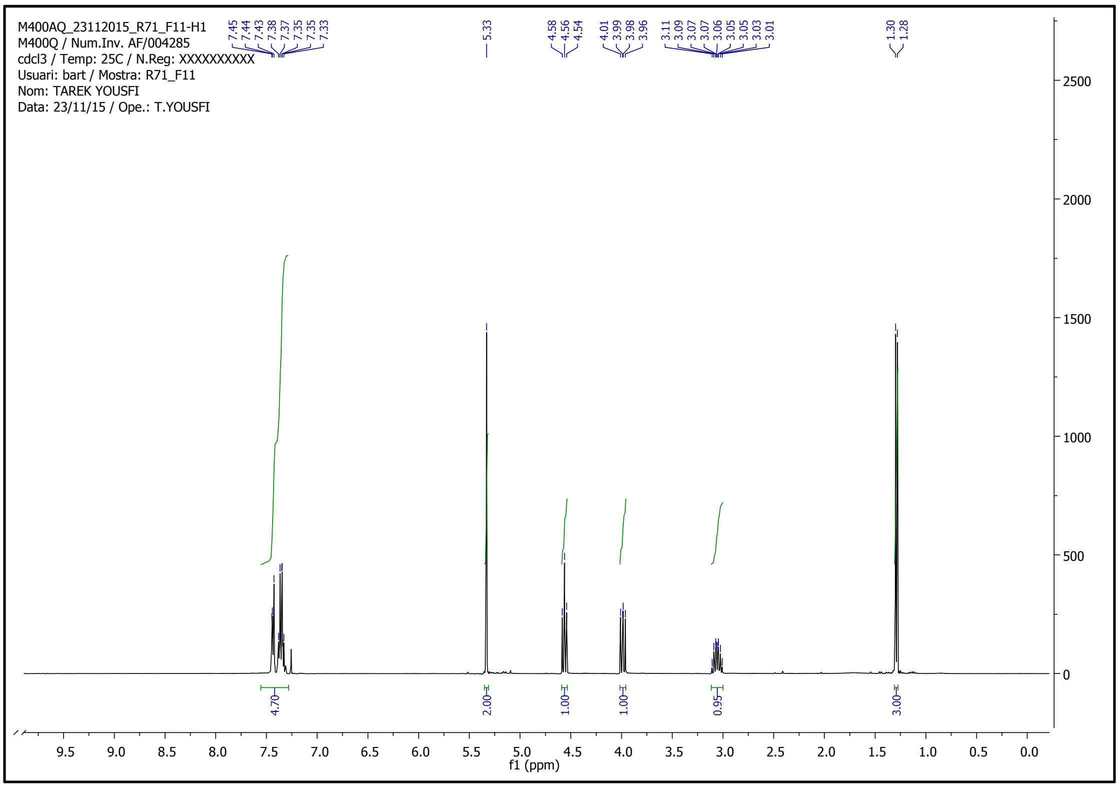

Figure S51. ^{1}H-NMR (400 MHz) of Benzyl 4-Methyl-5-oxo-isoxazolidine-2-carboxylate, **12a**.

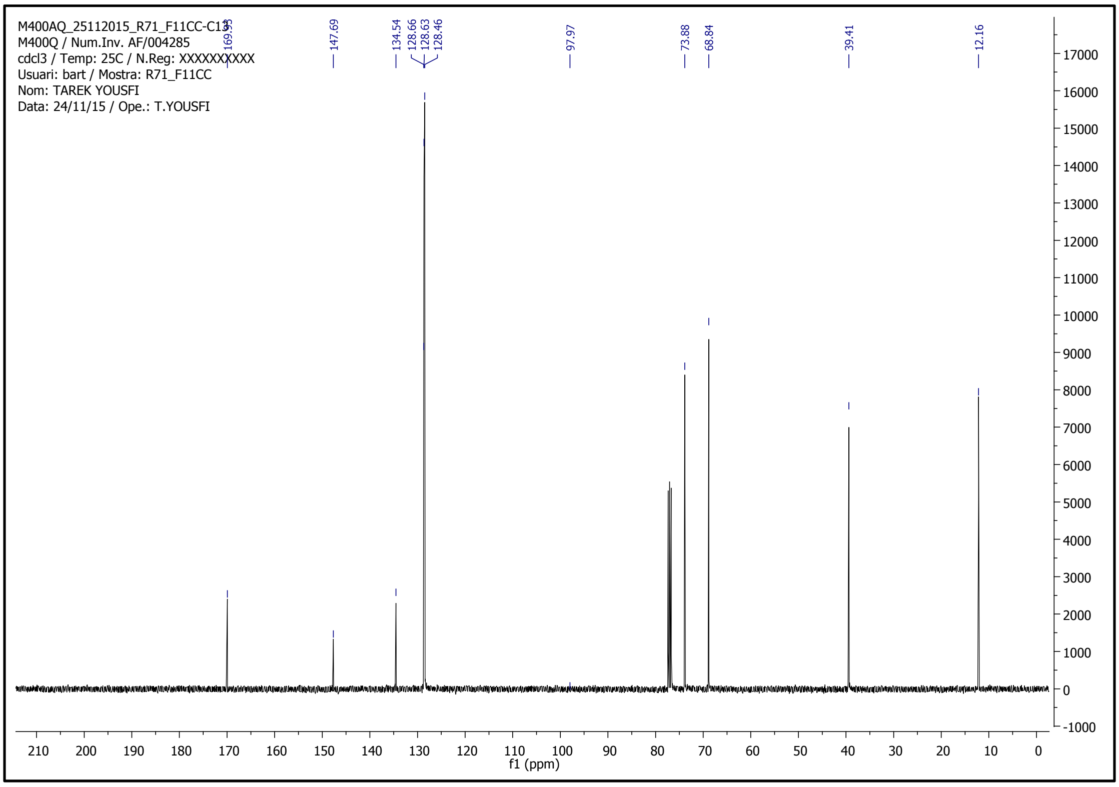

Figure S52. ^{13}C-NMR (100.6 MHz) of Benzyl 4-Methyl-5-oxo-isoxazolidine-2-carboxylate, **12a**.

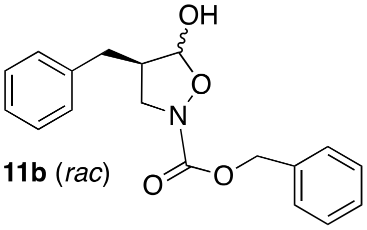

Figure S53. Benzyl 4-Benzyl-5-Hydroxy-isoxazolidine-2-carboxylate, **11b**.

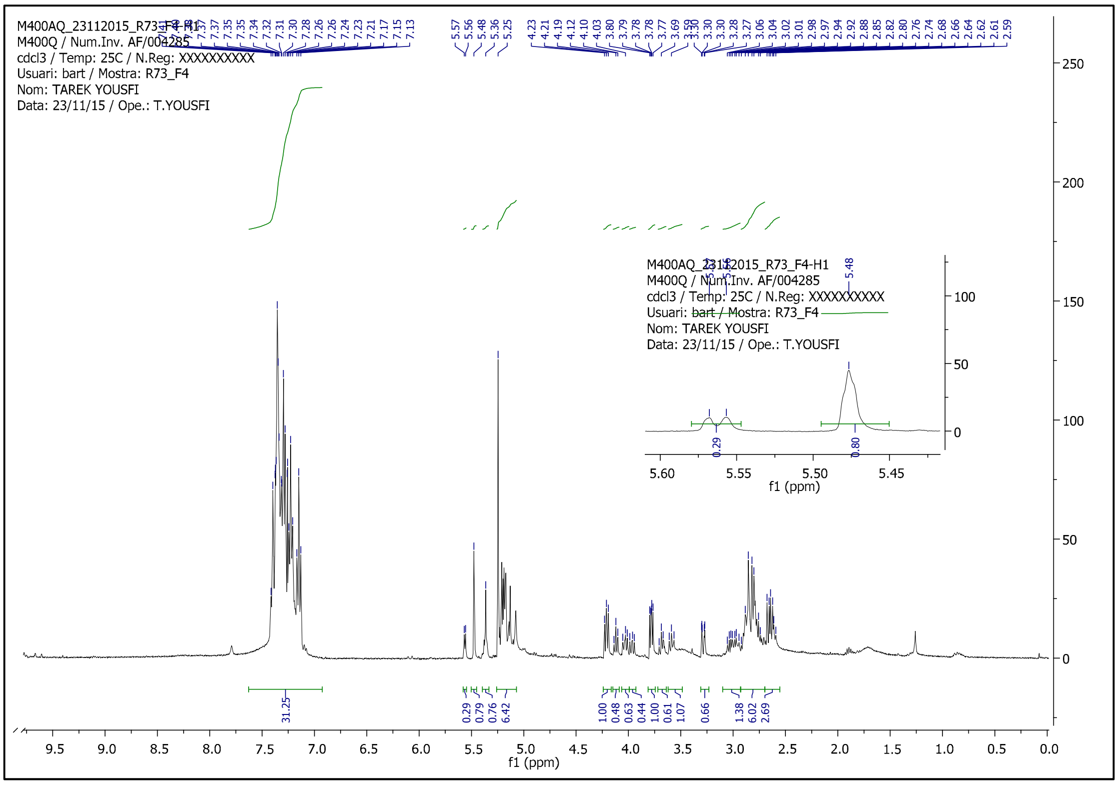

Figure S54. ^{1}H-NMR (400 MHz) of Benzyl 4-Benzyl-5-Hydroxy-isoxazolidine-2-carboxylate, **11b**.

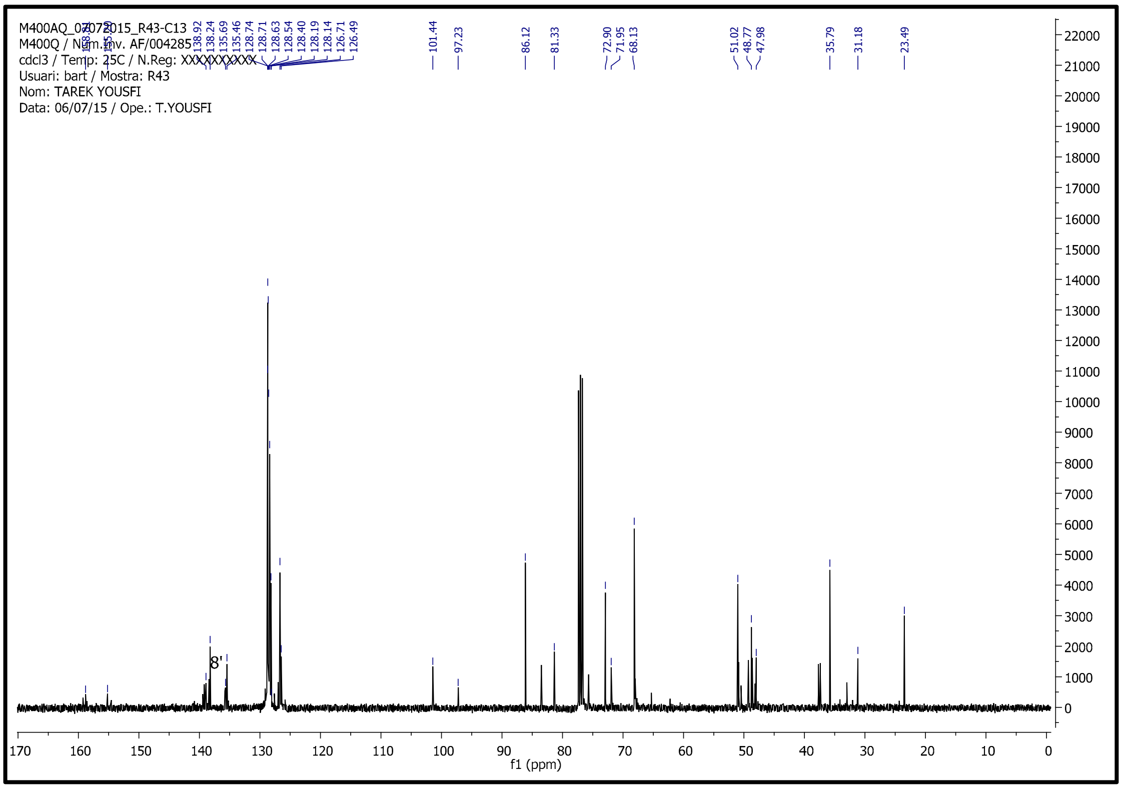

Figure S55. ^{13}C-NMR (100.6 MHz) of Benzyl 4-Benzyl-5-Hydroxy-isoxazolidine-2-carboxylate, **11b**.

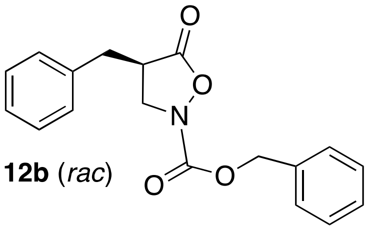

Figure S56. Benzyl 4-Benzyl-5-oxo-isoxazolidine-2-carboxylate, **12b**.

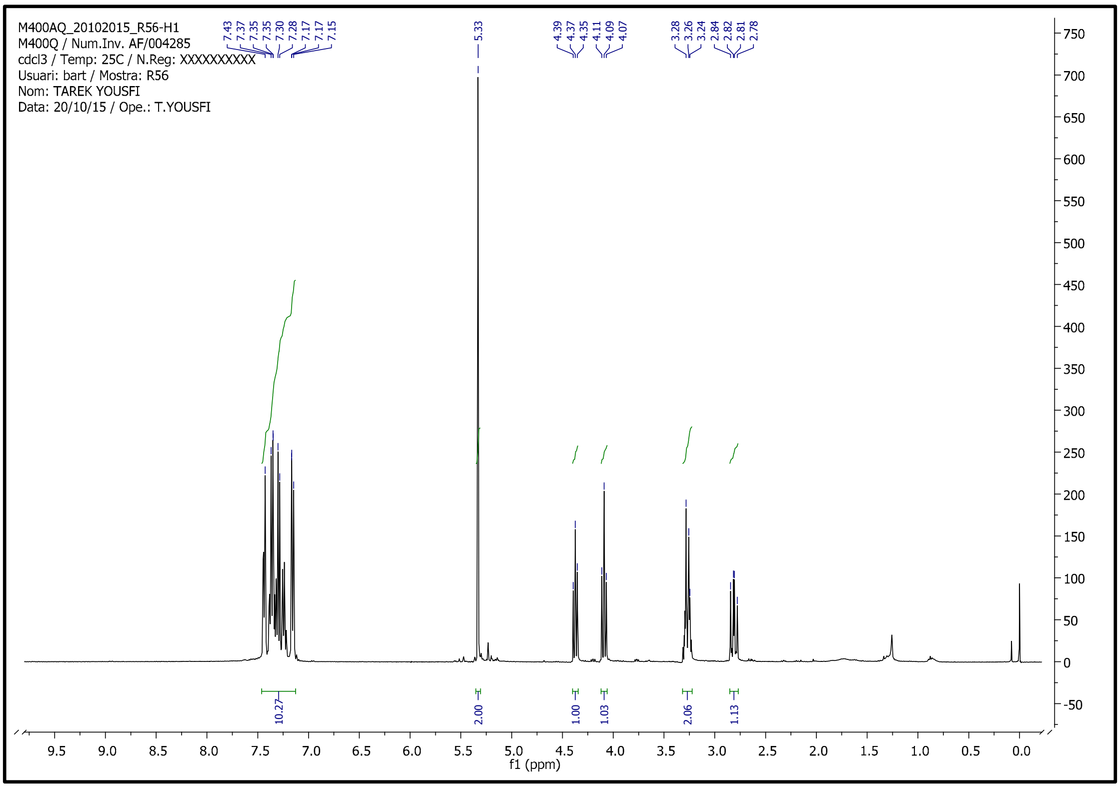

Figure S57. ^{1}H-NMR (400 MHz) of Benzyl 4-Benzyl-5-oxo-isoxazolidine-2-carboxylate, **12b**.

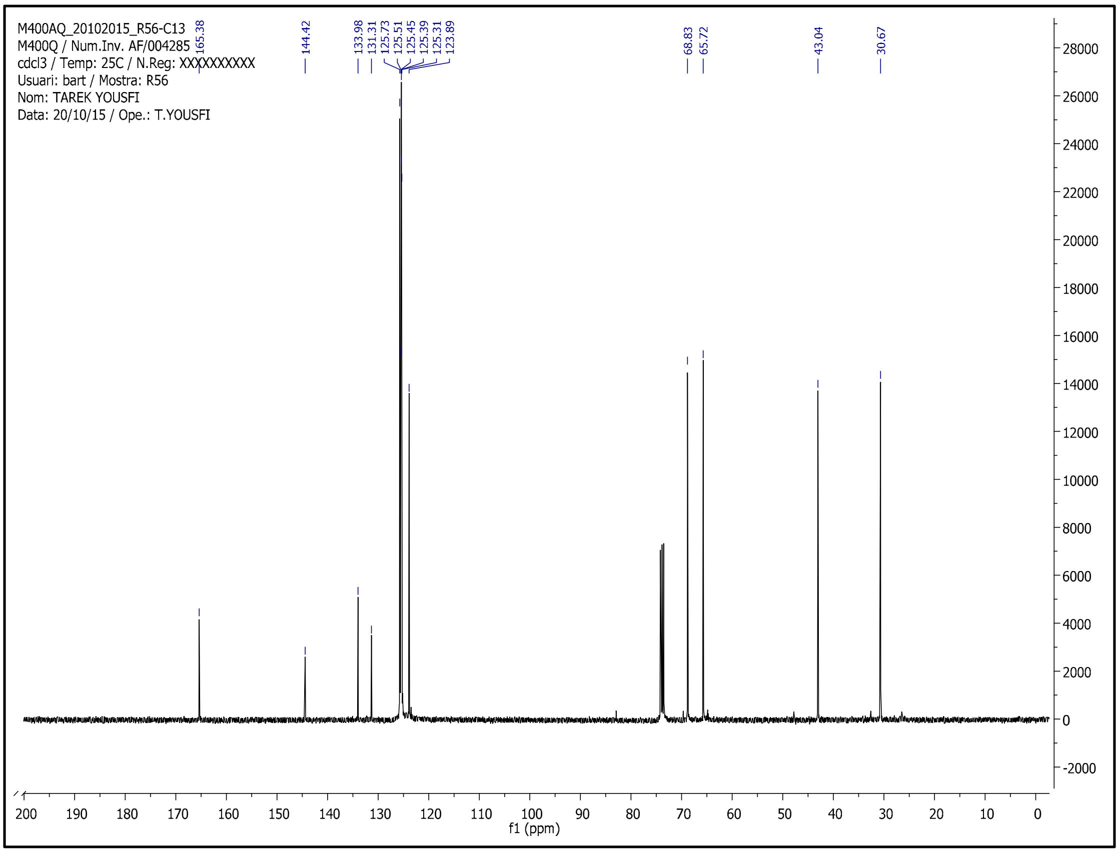

Figure S58. ^{13}C-NMR (100.6 MHz) of Benzyl 4-Benzyl-5-oxo-isoxazolidine-2-carboxylate, **12b**.

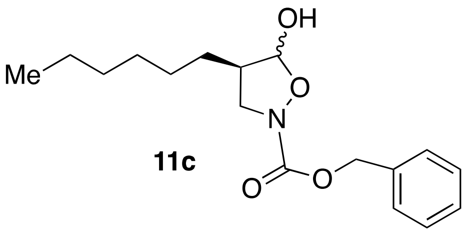

11c

Figure S59. Benzyl 4-Hexyl-5-hydroxy-isoxazolidine-2-carboxylate, **11c**.

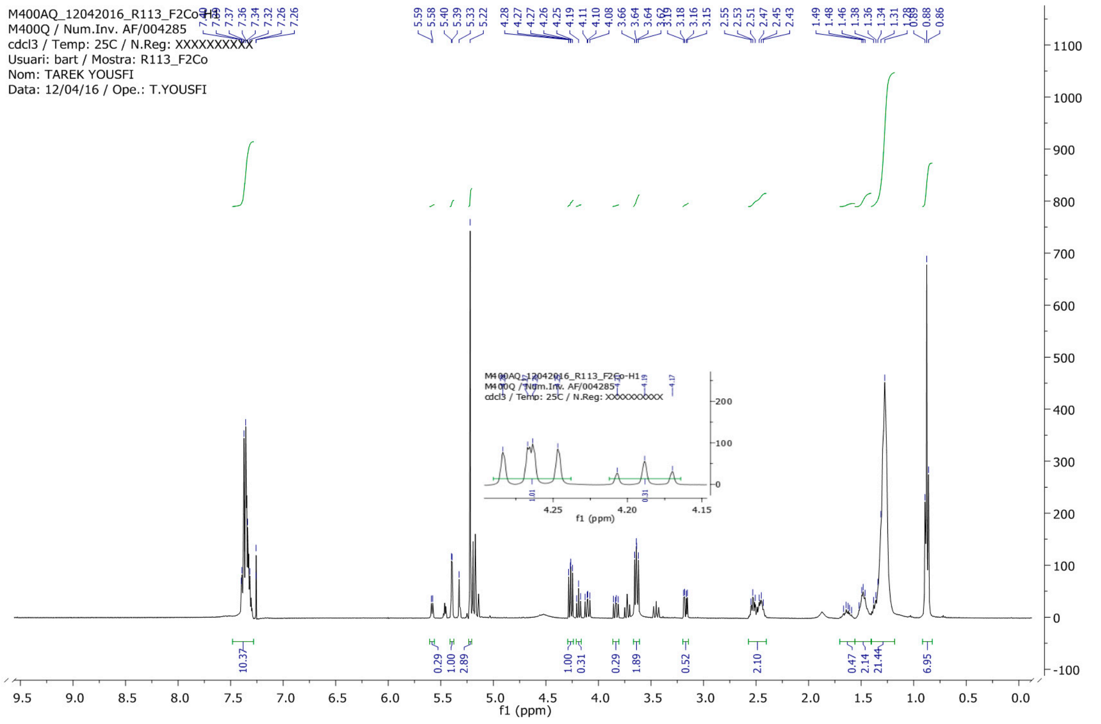

Figure S60. ^{1}H-NMR (400 MHz) of Benzyl 4-Hexyl-5-hydroxy-isoxazolidine-2-carboxylate, **11c**.

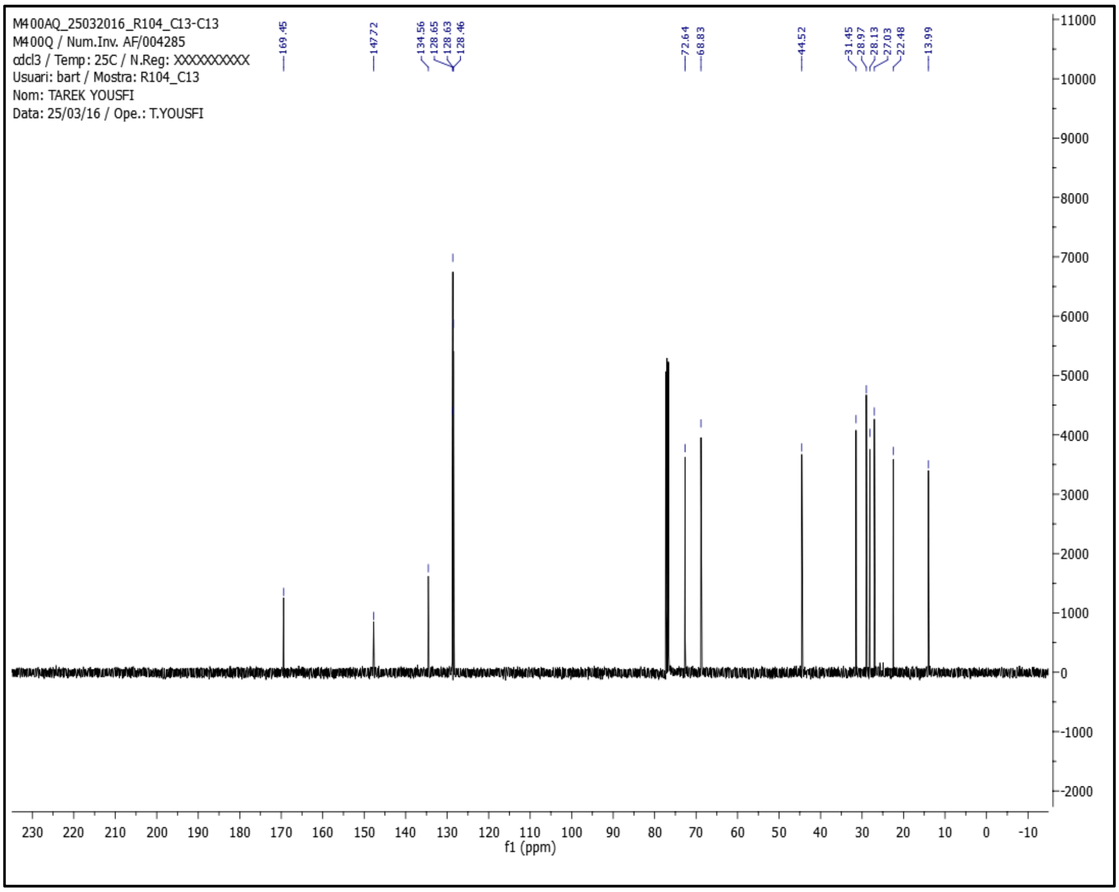

Figure S61. ^{13}C-NMR (100.6 MHz) of Benzyl 4-Hexyl-5-hydroxy-isoxazolidine-2-carboxylate, **11c**.

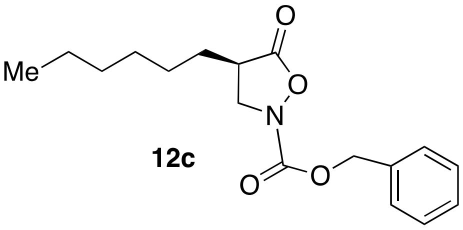

Figure S62. (−)-Benzyl 4-Hexyl-5-oxo-isoxazolidine-2-carboxylate, **12c**.

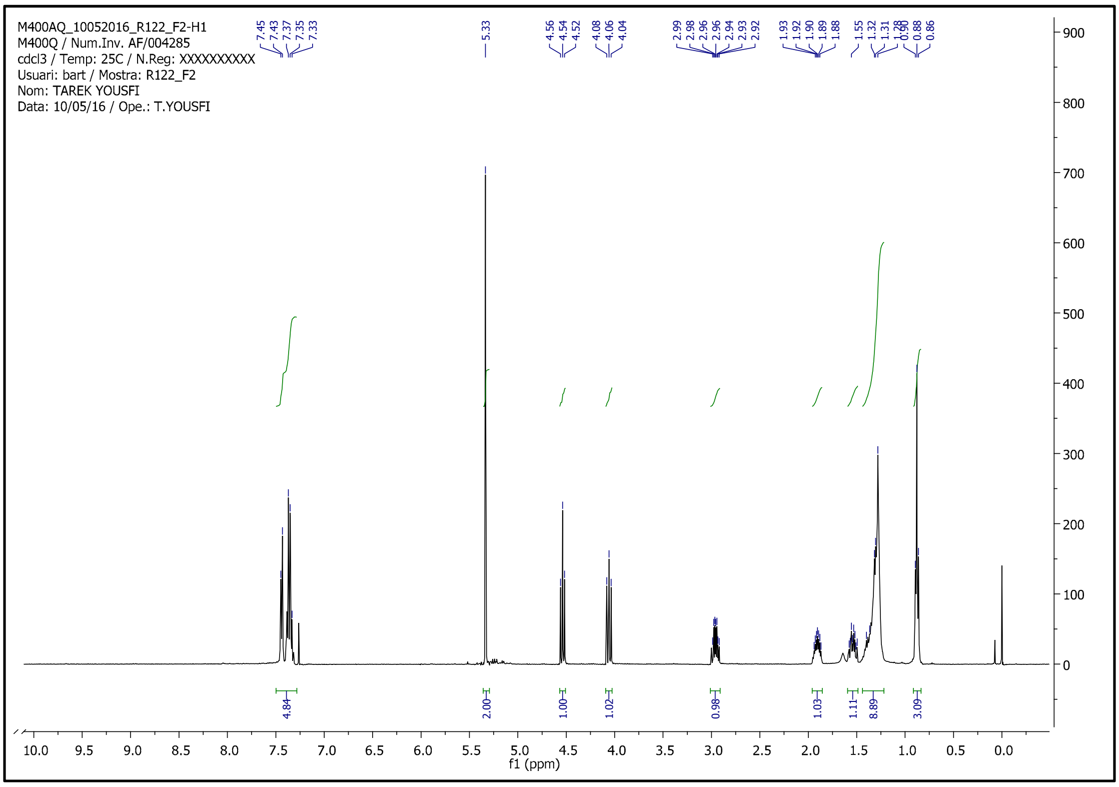

Figure S63. ^{1}H-NMR (400 MHz) of (−)-Benzyl 4-Hexyl-5-oxo-isoxazolidine-2-carboxylate, **12c**.

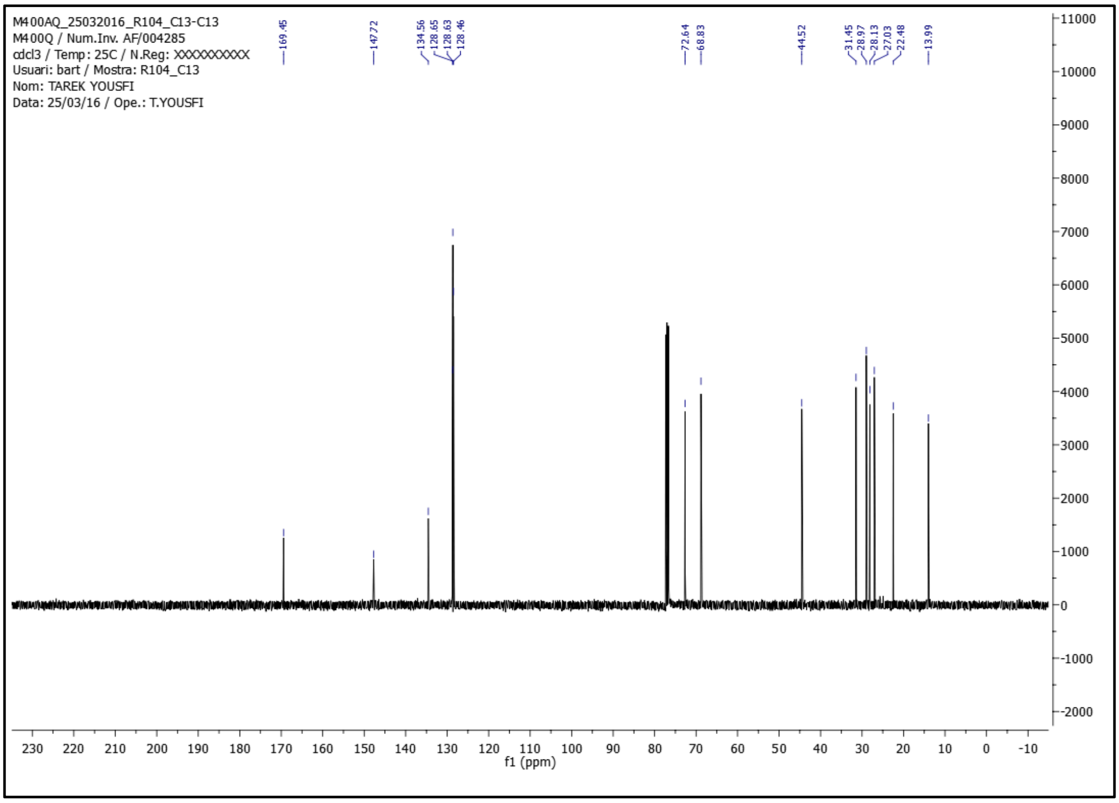

Figure S64. ^{13}C-NMR (100.6 MHz) of (−)-Benzyl 4-Hexyl-5-oxo-isoxazolidine-2-carboxylate, **12c**.

HPLC (Chiralpak® IB column, 90:10 hexane/isopropyl alcohol, 1 µL/min, λ = 254 nm, 25 °C).

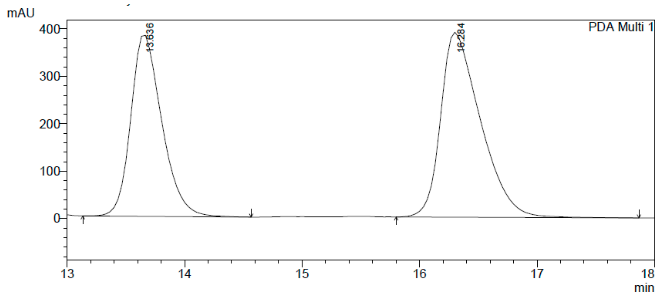

PeakTable

PDA Ch3 254nm 4nm

Peak#	Ret. Time	Area	Height	Area %	Height %
1	13.636	1272551	69970	43.403	49.555
2	16.284	1659359	71226	56.597	50.445
Total		2931910	141196	100.000	100.000

Figure S65. *rac*-**12c**.

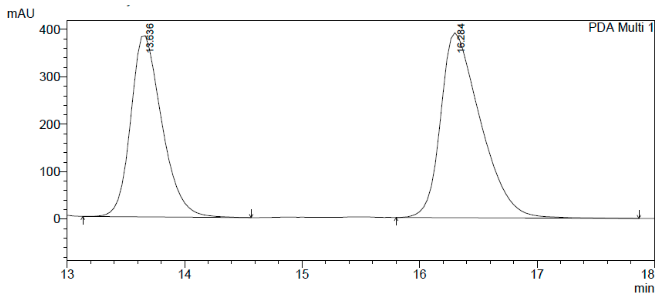

PeakTable

PDA Ch3 254nm 4nm

Peak#	Ret. Time	Area	Height	Area %	Height %
1	13.636	1272551	69970	43.403	49.555
2	16.284	1659359	71226	56.597	50.445
Total		2931910	141196	100.000	100.000

Figure S66. (−)-**12c**.
